# Secondary Organic Aerosol Generated from Biomass Burning Emitted Phenolic Compounds: Oxidative Potential, Reactive Oxygen Species, and Cytotoxicity

**DOI:** 10.1021/acs.est.3c09903

**Published:** 2024-04-29

**Authors:** Zheng Fang, Alexandra Lai, Dongmei Cai, Chunlin Li, Raanan Carmieli, Jianmin Chen, Xinming Wang, Yinon Rudich

**Affiliations:** † Department of Earth and Planetary Sciences, Weizmann Institute of Science, Rehovot 76100, Israel; ‡ Shanghai Key Laboratory of Atmospheric Particle Pollution and Prevention (LAP 3), Department of Environmental Science and Engineering, Fudan University, Shanghai 200438, China; § College of Environmental Science and Engineering, Tongji University, Shanghai 200072, China; ∥ Department of Chemical Research Support, Weizmann Institute of Science, Rehovot 76100, Israel; ⊥ State Key Laboratory of Organic Geochemistry and Guangdong Key Laboratory of Environmental Protection and Resources Utilization, Guangzhou Institute of Geochemistry, Chinese Academy of Sciences, Guangzhou 510640, China

**Keywords:** phenolic compounds, secondary organic aerosol, oxidative potential, reactive oxygen species, cytotoxicity

## Abstract

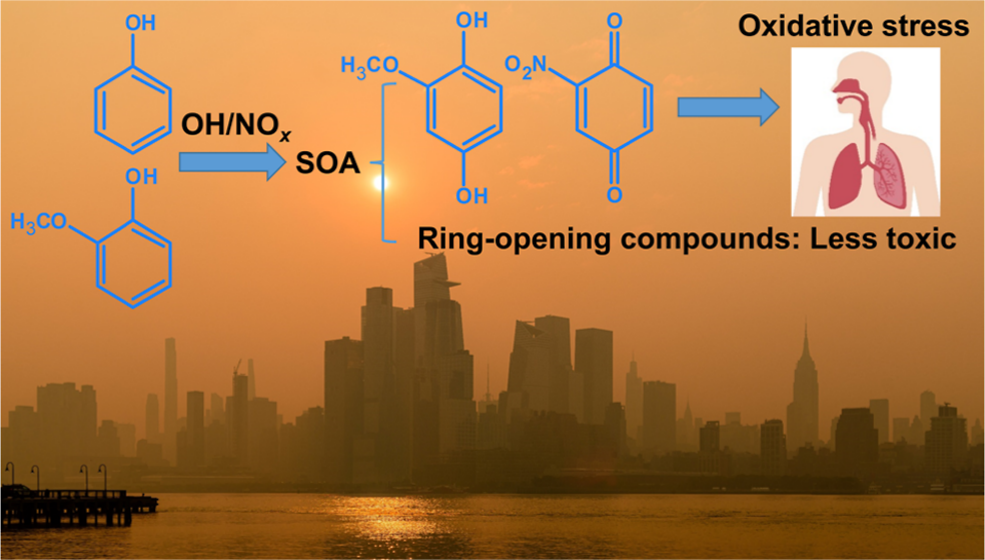

Phenolic compounds are largely emitted from biomass burning (BB) and have a significant potential to form SOA (Phc-SOA). However, the toxicological properties of Phc-SOA remain unclear. In this study, phenol and guaiacol were chosen as two representative phenolic gases in BB plumes, and the toxicological properties of water-soluble components of their SOA generated under different photochemical ages and NO_*x*_ levels were investigated. Phenolic compounds contribute greatly to the oxidative potential (OP) of biomass-burning SOA. OH-adducts of guaiacol (e.g., 2-methoxyhydroquinone) were identified as components of guaiacol SOA (GSOA) with high OP. The addition of nitro groups to 2,5-dimethyl-1,4-benzoquinone, a surrogate quinone compound in Phc-SOA, increased its OP. The toxicity of both phenol SOA (PSOA) and GSOA in vitro in human alveolar epithelial cells decreased with aging in terms of both cell death and cellular reactive oxygen species (ROS), possibly due to more ring-opening products with relatively low toxicity. The influence of NO_*x*_ was consistent between cell death and cellular ROS for GSOA but not for PSOA, indicating that cellular ROS production does not necessarily represent all processes contributing to cell death caused by PSOA. Combining different acellular and cellular assays can provide a comprehensive understanding of aerosol toxicological properties.

## Abstract 2

Phenol and guaiacol in biomass-burning smoke can form secondary organic aerosols that harm the human body via oxidative stress. Photochemical aging decreases toxicity, likely due to the breakdown of aromatic rings.

## 1 Introduction

In early June 2023, Canadian wildfires covered many North American cities in thick smoke, breaking records for air quality indices in New York and surrounding cities. As the smoke traversed the continent and turned the sky orange over vast populations, the health concerns of biomass burning (BB) events drew wide attention from governments, hospitals, and the academic community.^1^ Such an event is one of the ∼4.5 million global wildfires taking place every year. In recent decades, a growing wildfire trend has been observed in many parts of the world, coinciding with increased temperatures and drought severity.^2,3^ Apart from wildfires, wood, peat, and agricultural residues are frequently burned by humans, and these natural or anthropogenic processes are called BB. Several studies have indicated that BB exposure is associated with all-cause and cardiovascular mortality and respiratory morbidity.^4,5^

The dominant chemical components of biomass include cellulose, hemicellulose, and lignin. Pyrolysis of lignin yields a large number of phenolic compounds,^6,7^ such as phenol, catechol, guaiacol (2-methoxyphenol), and syringol (2,6-dimethoxyphenol). Phenolic compounds comprise an important portion of volatile organic compounds (VOCs) from BB^8^ and are frequently used as tracers of BB smoke.^7,9,10^ Additionally, specific phenolic compounds can be generated through the atmospheric oxidation of aromatic compounds. For example, benzene and anisole can be oxidized to produce phenol and methoxyphenols, respectively, through the electrophilic addition of OH radical.^11,12^ During atmospheric aging, phenolic compounds have great potential to form secondary organic aerosols (SOA), with reported SOA yields ranging from 0.24 to 0.54.^6,13^ Therefore, SOA formed from phenolic compounds contributes substantially to biomass burning SOA (BB-SOA)^14−17^ and has significant impacts on the physicochemical properties of BB-SOA. Extensive studies on the chemical composition,^7,13,18−21^ light absorption properties,^22,23^ and hygroscopic properties^24^ of SOA formed from phenolic compounds (PhC-SOA) have been conducted. However, less attention has been paid to their possible health effects.

Specific gas-phase phenolic compounds, including phenol, catechol, and cresols, have been listed as hazardous air pollutants by the United States Environmental Protection Agency. After 48 h of exposure to a sublethal concentration of phenol, reactive oxygen species (ROS) accumulation and programmed cell death were observed in diatom cells.^25^ Specific compounds in PhC-SOA were also found to be toxic. For example, nitrophenols can lead to ROS buildup and death in two types of human lung cells.^26^ Hydroquinones and quinones are redox-active molecules that can stimulate the production of superoxide (O_2_^–^) in a pH-dependent manner, favoring stronger acidity.^27−29^ However, specific phenolic compounds also have antioxidant properties because they can stabilize ROS by donating a hydrogen atom, and the resulting phenolic radicals are relatively stable due to their resonance structures.^30^ As a common biomass-burning marker in the aerosol phase,^31^ syringaldehyde can scavenge peroxyl radicals and thus has the potential to counter oxidative stress.^32^ Fang et al.^33^ reported that phenolic compounds were the second most abundant identified species in VOCs evaporated from wood tar, and the SOA formed from such VOCs were not lethal to human lung epithelial cells in an environmentally relevant exposure. Consequently, the net oxidative potential (OP) and cytotoxicity of PhC-SOA are difficult to predict because of the counterbalancing effects of endogenous ROS and antioxidants.

The OH oxidation of phenolic compounds is initiated by two main pathways: (1) electrophilic addition of an OH radical to the benzene ring and (2) OH radical abstraction of an H atom from the benzene ring.^6,7^ Several intermediate radicals from both reaction channels can react with O_2_ or with NO/NO_2_.^18^ The substituted functional group on the benzene ring will influence the branching ratios of the products, and the photochemical age will determine the oxidation extent. Under low-NO_*x*_ conditions, hydroperoxides, polyols, ring-opening acids, highly oxidized multifunctional compounds (HOMs), and quinones were identified in PhC-SOA;^6,11,34−36^ while under high-NO_*x*_ conditions, nitro-substituted products are the main products.^18,20^ Therefore, the functional groups on the benzene ring of the VOC precursor, NO_*x*_ levels, and the photochemical age can affect the chemical composition of PhC-SOA and influence its possible health effects.

In this study, two representative phenolic VOCs, phenol and guaiacol, were separately used as precursors to produce SOA in a flow tube reactor under different photochemical ages and NO_*x*_ levels. The oxidative potential of Phc-SOA was characterized by a dithiothreitol (DTT) assay. The ability of SOA to generate H_2_O_2_ and ROS radicals in water was measured with a fluorometric assay and by electron paramagnetic resonance (EPR) spectrometry, respectively. In vitro experiments on human lung epithelial cells were conducted to explore the cytotoxicity of Phc-SOA and its potential to induce cellular ROS. The chemical fingerprint of Phc-SOA was analyzed in parallel with both online and offline mass spectrometry. This article aims to estimate the toxicity of the SOA generated from two phenolic compounds with both acellular and cellular approaches and explore the influence of atmospheric aging conditions.

## 2 Materials and Methods

### 2.1 Generation of SOA

OH-initiated oxidation of phenolic compounds was conducted in a Potential Aerosol Mass (PAM) reactor (Aerodyne Research Inc., USA). The PAM reactor was operated in OFR254-60 mode with the principles and operational procedures described elsewhere.^37−40^ At 22.5 ± 0.2 °C, solid-state phenolic compounds were put in an impinger, and a controllable nitrogen stream was used to introduce 4–18 mL min^–1^ of their vapors into the PAM reactor. The initial concentration of externally supplied O_3_ was ∼60 ppm, and the OH exposure was calculated with the toolkit developed by Peng et al.^41^ In experiments without NO_*x*_, photochemical ages of 0.5 days and 5.0 days were explored by assuming an ambient daily average OH concentration of 1.5 × 10^6^ molecules cm^–3^.^42^ With the reported reaction rate constants *k*_OH_ and *k*_O_3__ of guaiacol,^7^ the ratios of ozonolysis in guaiacol experiments were estimated to be less than 2%. The *k*_O_3__ of phenol was not available. In NO_*x*_-involved experiments, two levels of the (RO_2_+NO)/(RO_2_+HO_2_) ratio, 0.4 and 1.0, were investigated to represent low-NO_*x*_ and high-NO_*x*_ conditions, respectively. In total, ten different types of Phc-SOA were investigated, with their experimental parameters and abbreviations summarized in Table S1. The level of the O_3_-induced SOA was measured when the phenolic compound and O_3_ were introduced in the dark. In different phenol and guaiacol experiments, the mass proportions of the O_3_-induced SOA in total SOA were 0.6–1.5 and 0.5–1.5%, respectively. SOA was collected on 13 (TF-450, Pall) and 47 mm (JHWP04700, Omnipore) Teflon filters. A scanning mobility particle sizer (SMPS, TSI) monitored the particle size distribution and volume concentration during filter sampling. An aerodynamic aerosol classifier (AAC, Cambustion, UK) was used in tandem with SMPS to measure the effective density of SOA (Table 1), which was used to convert the volume concentration in SMPS into the mass concentration of SOA. The SOA yield is defined as the ratio of the mass of formed SOA to the mass of reacted phenolic compounds (Table S1). The reacted VOCs were calculated according to *k*_OH_ of phenol and guaiacol^7^ and OH exposure in each experiment (Text S1). The Phc-SOA collected on filters was extracted into water and then analyzed by an ultrahigh-performance liquid chromatography (UHPLC) system (Dionex UltiMate 3,000, Thermo Electron, Inc.) coupled with a Q-Exactive Orbitrap MS (Thermo Electron, Inc.). The separation of analytes was performed on an Acquity UPLC HSS T3 column (1.8 μm particle size, 100 × 2.1 mm; Waters, Milford, MA, USA) at a flow rate of 0.3 mL min^–1^. The mobile phase consisted of (eluent A) ultrapure water with 0.1% acetic acid and (eluent B) acetonitrile with 0.1% acetic acid. The electrospray ionization (ESI) source was operated in negative mode. Blank filters were extracted and analyzed in the same manner. More details about the chemical analysis can be found in Cai et al.^43^

**Table 1:** Physicochemical Parameters of Each Type of Phc-SOA

SOA type	SOA density	O/C	OS_c_	N/C	CHON (*O*/*N* ≥ 3, %)^a^	aromatics (%)^b^	organic peroxides (%)^c^	PANs (%)^d^
“0.5 days” PSOA	1.54 ± 0.02	0.68 ± 0.01	–0.13 ± 0.02	0.015 ± 0.004	/	23.5	4.0	/
“0.5 days, low NO_*x*_” PSOA	1.52 ± 0.01	0.73 ± 0.01	–0.06 ± 0.01	0.085 ± 0.023	1.7	15.6	3.3	3.9 ± 0.0
“5 days” PSOA	1.50 ± 0.00	1.13 ± 0.01	0.50 ± 0.02	0.009 ± 0.003	/	12.3	5.8	/
“5 days, low NO_x_” PSOA	1.63 ± 0.01	1.20 ± 0.01	0.71 ± 0.03	0.050 ± 0.017	NM^e^	NM	NM	1.2 ± 0.0
“5 days, high NO_x_” PSOA	1.63 ± 0.01	1.18 ± 0.01	0.62 ± 0.01	0.060 ± 0.018	7.1	14.8	3.0	2.7 ± 0.0
“0.5 days” GSOA	1.58 ± 0.02	0.90 ± 0.01	–0.17 ± 0.01	0.010 ± 0.003	/	20.0	3.4	/
“0.5 days, low NO_x_” GSOA	1.50 ± 0.01	1.00 ± 0.02	–0.03 ± 0.03	0.030 ± 0.015	20.1	24.9	1.5	1.6 ± 0.1
“5 days” GSOA	1.59 ± 0.02	1.16 ± 0.01	0.35 ± 0.02	0.009 ± 0.005	/	16.6	4.8	/
“5 days, low NO_*x*_” GSOA	1.64 ± 0.02	1.50 ± 0.04	1.25 ± 0.08	0.039 ± 0.015	NM	NM	NM	3.2 ± 0.3
“5 days, high NO_*x*_” GSOA	1.64 ± 0.01	1.51 ± 0.09	1.32 ± 0.19	0.041 ± 0.014	15.9	20	2.1	4.3 ± 0.4

a “CHON (O/N ≥ 3, %)” is the signal proportion of the assigned nitro compounds and organonitrates in UHPLC-Orbitrap MS. These compounds contain carbon, hydrogen, oxygen, and nitrogen, with an elemental ratio of O/N ≥ 3 in the molecule.^43^ It is noted that for Phc-SOA produced without NO_*x*_, the proportion of CHON (O/N ≥ 3), and PANs were not zero (e.g., 7.4% of CHON and 0.7% of PANs in “5 days” PSOA), which could be attributable to impurities present in the N_2_. Therefore, when calculating CHON and PANs, the NO_*x*_ involved SOA were further background-corrected with their counterpart non-NO_*x*_ SOA. Values used for background corrections are labeled as “/”.

b “Aromatics (%)” is the signal proportion of the assigned aromatic compounds in UHPLC-Orbitrap MS. These compounds have aromaticity equivalent (Xc) values larger than or equal to 2.5.^43^

c “Organic peroxides (%)” is the signal proportion of the tentatively assigned organic peroxides in UHPLC-Orbitrap MS. The molecules of tentatively assigned organic peroxides are summarized in Text S4.

d “PANs (%)” is the molar proportion of PANs in SOA measured with the Griess assay, and the SOA generated in NO_*x*_-involved conditions were background-corrected with their non-NO_*x*_ counterpart SOA.

e NM = Not measured.

### 2.2 OP Measurements of the Soluble SOA

The OP of Phc-SOA was measured by a dithiothreitol (DTT) assay. The procedures have been described in Fang et al.^33^ and Li et al.^38^ The deionized water was used to extract SOA, and then the undissolved components were filtered by a syringe filter (SLLGC13NL, Millex-LG). With a total organic carbon analyzer (TOC-VCPH, Shimadzu) and a high-resolution time-of-flight aerosol mass spectrometer (HR-TOF-AMS, Aerodyne) to quantify OC concentration and the OM/OC ratio, respectively, the concentration of the soluble SOA can be obtained, which was controlled to be within 50–70 mg L^–1^. The extraction efficiency for SOA under all conditions had an average value of 0.88. 0.5 mL portion of aerosol extract solution, 4 mL of sodium phosphate buffer (0.1 M, pH = 7.4), and 0.5 mL of DTT solution (1 mM) were added to an incubation vial, which was kept at 37 °C in a dry bath incubator (MD-01N, MRC). Every 5 min, 0.5 mL of the solution in the incubation vial is withdrawn and mixed with 1.5 mL of Tris base solution (0.4 M), 0.5 mL of TCA solution (10 w/v %), and 0.5 mL of DTNB solution (1 mM) to form 2-nitro-5-thiobenzoic acid, which has a light absorption peak at 412 nm and can be characterized by UV–vis spectroscopy (model USB 650, Ocean Optics). Approximately 7–9 data points were obtained before 30% of DTT was consumed and were linearly fitted versus time to determine the consumption rate of DTT by the SOA solution. Blank filters were analyzed by the same procedures to obtain the background value of the DTT decay rate. Duplicate OP measurements were conducted for both the SOA and blank samples. The DTT consumption rate (σ^DTT^, mM min^–1^) and the mass-normalized DTT activity (OP^DTT^, pmol min^–1^ μg^–1^) of a sample were calculated as follows
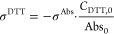
1
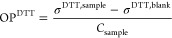
2where σ^Abs^ (min^–1^) is the decay rate of 2-nitro-5-thiobenzoic acid absorbance versus the incubation time, Abs_0_ is the initial absorbance of 2-nitro-5-thiobenzoic acid absorbance extrapolated from the linear regression, *C*_DTT,0_ (mM) is the initial DTT molar concentration in the reaction cuvette, and *C*_sample_ (mg L^–1^) is the mass concentration of a sample in the reaction cuvette. The OP for operational blank and for 1,4-naphthoquinone as positive controls were measured frequently to ensure the reproducibility of the DTT assay. The measured OP for 1,4-NQ [Figure S10, (3.83 ± 0.20) × 10^3^ pmol min^–1^ μg^–1^] was stable throughout the campaign and was consistent with previous studies.^38,44^

### 2.3 Quantification of ROS in Water Solution: Radicals and H_2_O_2_

The ability of Phc-SOA to generate ROS radicals in water was measured by a Bruker ELEXSYS E500 X-band electron paramagnetic resonance (EPR) spectrometer equipped with a Bruker ER4102ST resonator in a Wilmad flat cell (WG-808-Q) for 100 scans at room temperature. The setup parameters for the EPR spectrometer used in this study were the same as those in Chowdhury et al.^45^ 5-*tert*-Butoxycarbonyl-5-methyl-1-pyrroline-*N*-oxide (BMPO, Enzo Life sciences) was used to transfer ROS radicals to adducts that remain paramagnetic. The BMPO adducts are more stable than their parent radicals and can exhibit distinct EPR spectral patterns according to the ROS type.^46,47^ Briefly, filters with Phc-SOA were extracted with a water solution of 4.5 g L^–1^ BMPO by vortex shaking for 10 min and were then immediately analyzed by the EPR spectrometer. As shown in Figure 2, ROS radicals were detected in 6 out of 10 Phc-SOA. In these 6 experiments, an OH scavenger, dimethyl sulfoxide (DMSO), was used to distinguish OH radicals.^48^ SOA filters were extracted with 4.5 g L^–1^ of BMPO dissolved in 10% (in volume) DMSO + 90% H_2_O solution for 10 min. Differences in EPR spectra with and without DMSO were used to quantify OH radicals. The calibration curve was acquired based on known concentrations (0, 10, 20, 40, and 80 μM) of a stable nitroxide radical, 3-carboxy-proxyl (Figure S3a). All of the EPR measurements were performed in duplicate.

The Fluorometric Hydrogen Peroxide Assay Kit (MAK165, Sigma) was used to measure the H_2_O_2_ yield of SOA. The kit utilizes a peroxidase substrate that generates a red fluorescent product after the reaction with hydrogen peroxide. To remove the interference from organic peroxides in SOA, the SOA water extracts were measured with and without catalase to selectively quantify H_2_O_2_ (Text S2). As potential precursors of ROS in SOA solution, peroxyacyl nitrates (PANs) were measured with the Griess assay. Operational procedures to measure the PANs are described in Text S3.

The molar yield (*Y*_ROS_) of ROS species from SOA is calculated as follows
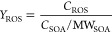
3where *C*_ROS_ (mmol L^–1^) is the molar concentration of ROS species measured by EPR, or the Fluorometric Hydrogen Peroxide Assay Kit, *C*_SOA_ (mg L^–1^) is the mass concentration of soluble SOA in water, and MW_SOA_ is the average molar mass of SOA, which is assumed to be 200 g mol^–1^. Typical *C*_SOA_ for ROS measurement was 50–70 mg L^–1^ to keep consistent with the DTT assay.

### 2.4 Cell Exposure

The biological effects of Phc-SOA and chemical standards were tested in vitro at varying concentrations between 10 and 800 μg/mL in human alveolar epithelial cells from the lung adenocarcinoma cell line A549 (ATCC). A549 cells were cultured in RPMI-1640 medium (Gibco, Thermo Fisher Scientific, USA) supplemented with 5 μg/mL penicillin/streptomycin, 2 mM glutamine, and 10% fetal bovine serum (Biological Industries, Beit Ha-Emek, Israel) in a humidified incubator at 37 °C and with 5% CO_2_. Cells were dissociated from tissue culture flasks using 0.25% trypsin–EDTA (Gibco), resuspended in culture media, and seeded in 12-well plates at a density of 1.5 × 10^5^ cells/well 1 day before exposures.

Buffered SOA extracts for cell exposures were prepared by extracting filters in a salts glucose medium (SGM) consisting of 500 mM HEPES, 1 M NaCl, 50 mM KCl, 20 mM CaCl_2_, and 50 mM dextrose at pH 7.2. Chemical standards were dissolved in either SGM (hydroquinones and nitrophenols) or, when necessary for solubility, SGM with 0.5% DMSO (quinones). Blank controls consisted of either clean filters extracted in the same manner (for SOA samples) or SGM (with or without DMSO, corresponding to chemical standards). Cell death and cellular ROS were measured using propidium iodide and 2′,7′-dichlorofluorescein diacetate (DCFH-DA), respectively, and their procedures are in Text S5. All exposures were conducted in triplicate wells and repeated at least two times. Flow cytometry data were collected from at least 10,000 cells.

## 3 Results and Discussion

### 3.1 Bulk Chemical Composition of Phc-SOA

Elemental ratios of Phc-SOA were obtained from AMS (Table 1). With a photochemical age of 0.5 days and in the absence of NO_*x*_, phenol SOA (PSOA) and guaiacol SOA (GSOA) had an O/C ratio of 0.68 ± 0.01 and 0.90 ± 0.01, respectively. High O/C ratios indicate that highly oxygenated products of multigeneration reactions are important components of Phc-SOA.^6^ The oxidation states of carbon (OS_c_) of the “0.5 days” PSOA and GSOA were −0.13 ± 0.02 and −0.17 ± 0.01, respectively, which are classified as “less-oxidized oxygenated OA” in ambient aerosols (LO-OOA).^49,50^ Upon further aging, the O/C ratio and OS_c_ values increased as expected. The presence of NO_*x*_ increased the N/C ratio of Phc-SOA from AMS measurement, and UHPLC-Orbitrap MS also observed a larger proportion of nitro compounds and organonitrates in NO_*x*_-involved conditions (Table 1).

### 3.2 OP of Water-Soluble Phc-SOA

**Figure 1:**
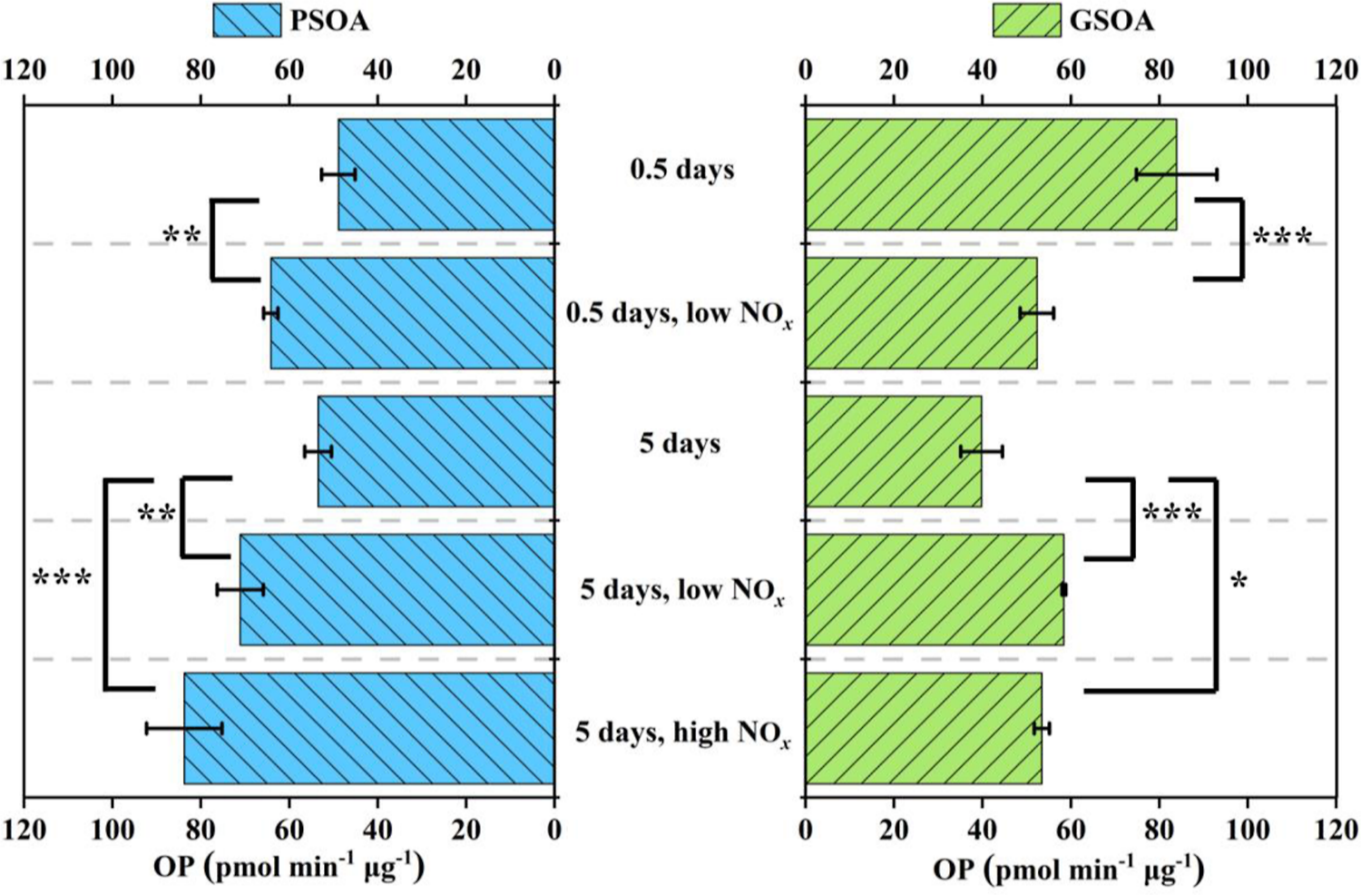
OP^DTT^ of Phc-SOA under different aging conditions. Comparisons between NO_*x*_ involved and non-NO_*x*_ conditions were conducted with an analysis of variance (ANOVA). The symbols “***”, “**”, “*”, “ns” mean *p* ≤ 0.005, *p* ≤ 0.01, *p* ≤ 0.05, and *p* > 0.05, respectively.

The OP values of PSOA and GSOA formed at different oxidation conditions and of reference BB-related SOA are summarized in Figures 1 and S4. The OP of Phc-SOA ranged between 39.8 and 83.9 pmol min^–1^ μg^–1^, which is lower than the OP of naphthalene SOA^51^ but is comparable to or higher than the rest of BB-related SOA shown in Figure S4.

The effect of NO_*x*_ on the OP of aromatic SOA is specific to its VOC precursor and to the aging condition. For the PSOA, the addition of NO_*x*_ leads to higher OP values for both “0.5 days” and “5 days” conditions (*p* ≤ 0.05). For the “5 days” GSOA, NO_*x*_ addition led to higher OP (*p* ≤ 0.05), while the “0.5 days” GSOA has the highest OP (83.9 ± 9.1 pmol min^–1^ μg^–1^) among all conditions, and the addition of NO_*x*_ decreased the OP to 52.3 ± 3.8 pmol min^–1^ μg^–1^. Upon NO_*x*_ addition, Tuet et al.^52^ observed increases in OP for naphthalene SOA but no change for *m*-xylene SOA; Jiang et al.^53^ found that the OP of toluene SOA and 1,3,5-trimethylbenzene SOA were not sensitive to NO_*x*_; Li et al.^38^ reported decreased OP for anisole SOA under high NO_*x*_ levels.

It has been reported that the first-generation phenolic compounds have a molar yield of 0.27–0.31.^20^ In our study, higher OP values were observed in relatively fresh GSOA, so the OP of several early-generation products (Table 2) in Phc-SOA were explored. Hydroquinone, catechol, 4-nitrophenol, and 2-nitrophenol were representative ring-retaining early-stage products in PSOA, and no detectable OP was measured for these chemical standards. 4-Nitroguaiacol and 2-methoxyhydroquinone are representative ring-retaining early-stage products in GSOA, and 4-nitroguaiacol is inert to DTT, while 2-methoxyhydroquinone has an OP of 125.8 ± 1.7 pmol min^–1^ μg^–1^. In the presence of NO_*x*_, the formation of 2-methoxyhydroquinone and its further oxidation products is in competition with the formation of nitro-containing compounds. The standard mass spectrum of 2-methoxyhydroquinone in UHPLC-Orbitrap MS presents two characteristic ions of C_6_H_3_O_3_^–^ and C_7_H_7_O_3_^–^ (Figure S5), and their summed signal proportion in the “0.5 days” GSOA was 21%. Upon photochemical aging conditioned to 0.5 days, the presence of low NO_*x*_ decreased the mass-normalized signals of C_6_H_3_O_3_^–^ and C_7_H_7_O_3_^–^ in generated SOA by 21 and 37%, respectively. This partly explains why the “0.5 days” GSOA has a relatively high OP. A decrease in the GSOA OP was also observed when its photochemical age increased from 0.5 to 5.0 days. This is consistent with a decrease in the content of early-stage products with a similar structure to 2-methoxyhydroquinone. In the UHPLC-Orbitrap MS, the mass-normalized signals of C_6_H_3_O_3_^–^ and C_7_H_7_O_3_^–^ decreased by 77 and 84%, respectively, from the “0.5 days” GSOA to the “5 days” GSOA.

**Table 2:** DTT Activity of Chemical Standards in Water Solution

organic species	concentration (g L^–1^)	OP (pmol min^–1^ μg^–1^)
1,4-hydroquinone	0.10	LDL
catechol	0.15	LDL
4-nitrophenol	0.10	LDL
2-nitrophenol	0.15	LDL
2-methoxyhydroquinone	0.10	125.8 ± 1.7
4-nitroguaiacol	0.10	LDL
1,4-dihydroxy-2,6-dimethoxybenzene	0.02	250.2 ± 12.5
2,5-dimethyl-1,4-benzoquinone	0.01	99.2 ± 0.8^a^
2,5-dimethyl-3,6-dinitro-1,4-benzoquinone	0.01	620.7 ± 29.4

a With 2,5-dimethyl-1,4-benzoquinone, DTT decays much faster during 0–20 min than during 20–40 min. The average DTT consumption rate during 0–40 min was derived with the solution absorbance measured at the beginning (0.5 min) and end (40 min) of the experiment rather than doing a linear fitting for the whole period.

It is interesting that hydroquinone causes no decay of DTT, while 2-methoxyhydroquinone has a relatively high OP. To confirm the inference that the methoxy group can increase the OP of hydroquinone, we measured the OP of 1,4-dihydroxy-2,6-dimethoxybenzene, which has an additional methoxy group compared with 2-methoxyhydroquinone, and indeed, it has a higher OP (250.2 ± 12.5) than 2-methoxyhydroquinone. It is noted that 1,4-dihydroxy-2,6-dimethoxybenzene is the OH-adduct of syringol, which is also a representative biomass pyrolysis product. Therefore, fresh syringol SOA formed in the daytime may also have a considerable OP in the water solution. A proposed mechanism of how 2-methoxyhydroquinone catalyzes the consumption of DTT is depicted in Figure S6a. First, DTT is oxidized by O_2_ dissolved in water to form O_2_^–^·. The OP of pure water, which serves as a negative control for all SOA measurements, exhibits a negligible DTT decay rate of 7.15–7.62 × 10^–5^ mM min^–1^. Second, the 2-methoxyhydroquinone is oxidized by O_2_^–^· to the semiquinone radical.^54^ Compared to hydroquinone, the methoxy group in 2-methoxyhydroquinone is electron-donating and can stabilize the semiquinone radical, thus facilitating this reaction. Then, the semiquinone radical catalyzes the reaction between DTT and O_2_ by first donating electrons to O_2_ and then obtaining electrons from DTT. The O_2_^–^· formed in reaction 3 can also participate in reaction 2 to rapidly transform 2-methoxyhydroquinone into its semiquinone.

We observed mixed trends in how NO_*x*_ affects the OP: for the “0.5 days” GSOA, NO_*x*_ interfered with the formation of 2-methoxyhydroquinone and its OH-adducts, which partly explains the decrease in OP; however, for PSOA at both photochemical ages and for GSOA at a photochemical age of 5 days, the OP increased with the NO_*x*_ addition. Quinones are photochemical reaction products of phenolic compounds.^6,11,34^ Illustrated by this and also limited by the commercial availability of quinone compounds, 2,5-dimethyl-3,6-dinitro-1,4-benzoquinone (MNQ) and 2,5-dimethyl-1,4-benzoquinone (MQ) were chosen to represent quinones formed with and without NO_*x*_, respectively and the OP of their chemical standards were investigated. 1,4-Hydroquinone and 2-methoxy-1,4-hydroquinone were proposed products in PSOA and GSOA under the non-NO_*x*_ condition, respectively.^6,18,34^ Compared to 1,4-hydroquinone, the surrogate quinone compounds used here have two more methyl groups. The methyl group has a relatively weak electron-donating ability, which can help stabilize the semiquinone radical generated during its redox reaction with DTT. Therefore, the OP^DTT^ of surrogate quinone compounds is presumably larger than their counterpart compounds in PSOA. As shown in Table 2, adding two nitro groups to MQ turns it into MNQ, and the OP increases from 99.2 ± 0.8 to 620.7 ± 29.4. As an electron-withdrawing group, a nitro group should decrease the stability of semiquinone, which is opposite the methoxy group. Therefore, it is proposed in Figure S6b that the nitro group may directly obtain electrons from DTT to form the anion radical (NO_2_^–^·) and then reduces O_2_ to O_2_^–^· and regenerates its parental structure.^55^ In this way, the addition of a nitro group to quinones shall increase the OP in specific cases. Apart from this, the ring-opening products of specific nitro-aromatics may be electron-deficient alkenes (Figure S6c) and can consume DTT through the Michael addition reaction.^56^

### 3.3 ROS Yield of Phc-SOA in Water

**Figure 2:**
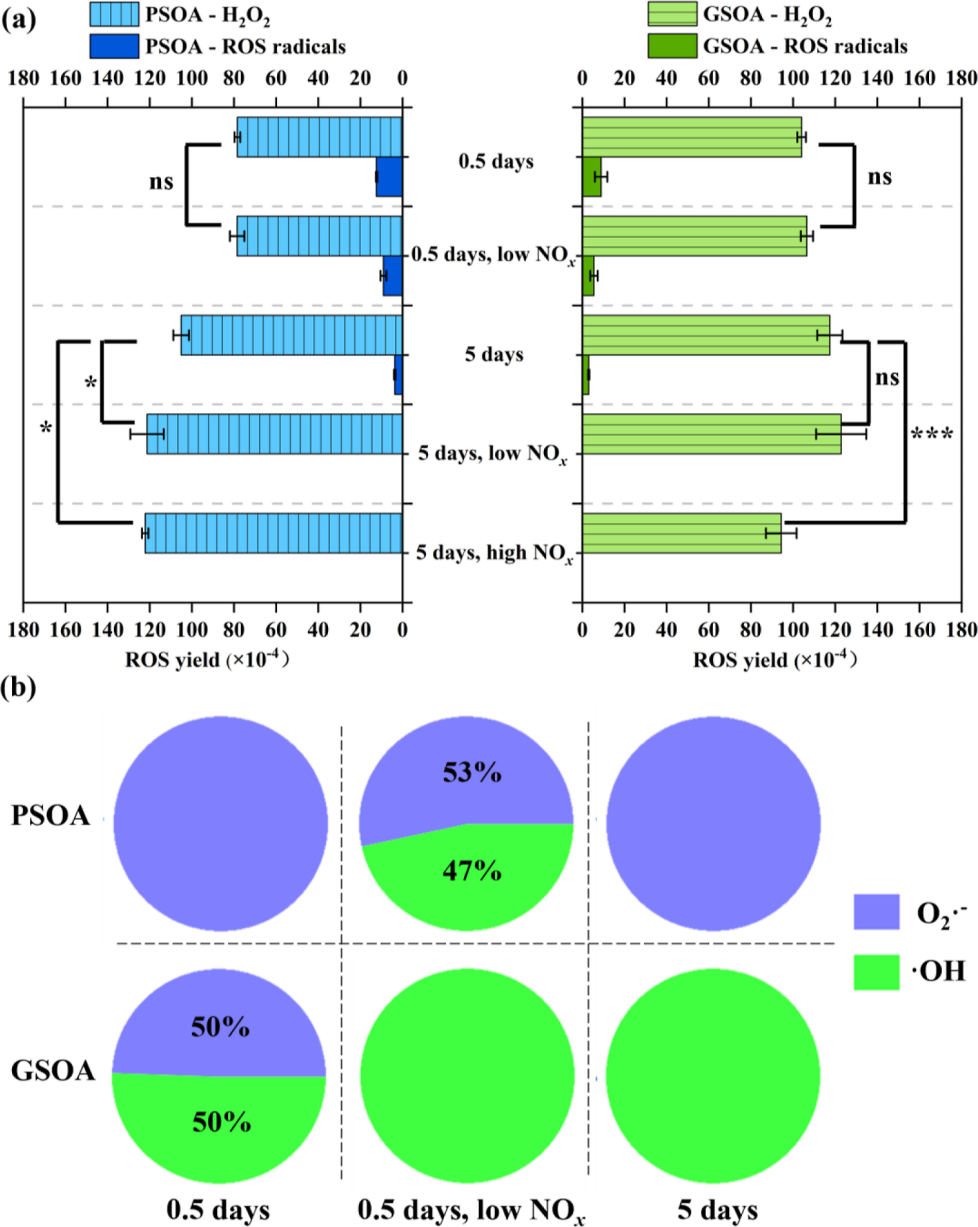
(a) Molar yield of ROS from Phc-SOA in water solution. Only the yield of H_2_O_2_ was compared. The symbols “***”, “**”, “*”, “ns” mean *p* ≤ 0.005, *p* ≤ 0.01, *p* ≤ 0.05, and *p* > 0.05, respectively. (b) Relative contents of O_2_·^–^ and ·OH produced by Phc-SOA where ROS radicals were detectable.

H_2_O_2_ is the main detected ROS species in the aqueous solution of Phc-SOA, as shown in Figure 2a. The molar yields of H_2_O_2_ are 0.78–1.22 × 10^–2^ and 0.94–1.23 × 10^–2^ for PSOA and GSOA, respectively, which are comparable to SOA generated from other aromatic VOCs. For example, toluene SOA and naphthalene SOA have H_2_O_2_ yields of 0.89 × 10^–2^ and 0.67–0.91 × 10^–2^, respectively.^27,28^ Based on the line shape in the EPR spectra, the ROS radicals were identified as ·OH and O_2_^–^·, and with DMSO as the OH scavenger, the contributions of ·OH and O_2_^–^· were quantified (Figure S11). O_2_^–^· and ·OH were the dominant radical species in PSOA and guaiacol SOA, respectively (Figure 2b). The molar yield of ROS radicals was lower than the detection limit for the “5 days” Phc-SOA in the presence of NO_*x*_. For the SOA in which ROS radicals were detectable, their total yields were about 1 order of magnitude smaller than H_2_O_2_. The dominance of H_2_O_2_ among produced ROS species in water has also been reported for naphthalene SOA and toluene SOA in a recent study.^27^ Semiquinones have been suggested to be the main source of superoxide in aromatic SOA through their reactions with O_2_ in water, followed by the recombination of superoxide radicals to form H_2_O_2_ molecules in the presence of H^+^.^27,28^ However, a much higher molar yield was observed for H_2_O_2_ than for superoxide, suggesting possible other sources of H_2_O_2_ in Phc-SOA. In the atmospheric oxidation processes of phenolic compounds, organic hydroperoxides are formed in the “RO_2_+HO_2_” pathway (Yee et al., 2013; Wang et al., 2017),^6,35^ while PANs are produced through the “RO_2_+NO” pathway. Both organic hydroperoxides and PANs can hydrolyze to form H_2_O_2_ in the liquid phase.^57−59^ As shown in Table 1, the presence of NO_*x*_ promoted the formation of PANs and inhibited organic peroxides, which is in line with theoretical expectations. The low NO_*x*_ condition has no significant (*p* > 0.05) impact on the H_2_O_2_ yield of the “0.5 days” Phc-SOA, which likely indicates the balancing of an increase in PANs and a decrease in organic hydroperoxides. At the photochemical age of 5 days, a high NO_*x*_ level increased the H_2_O_2_ yield of PSOA but decreased that of GSOA, implying that the influence of NO_*x*_ is both dose- and precursor-dependent.

### 3.4 In Vitro Toxicity of Chemical Standards

**Figure 3:**
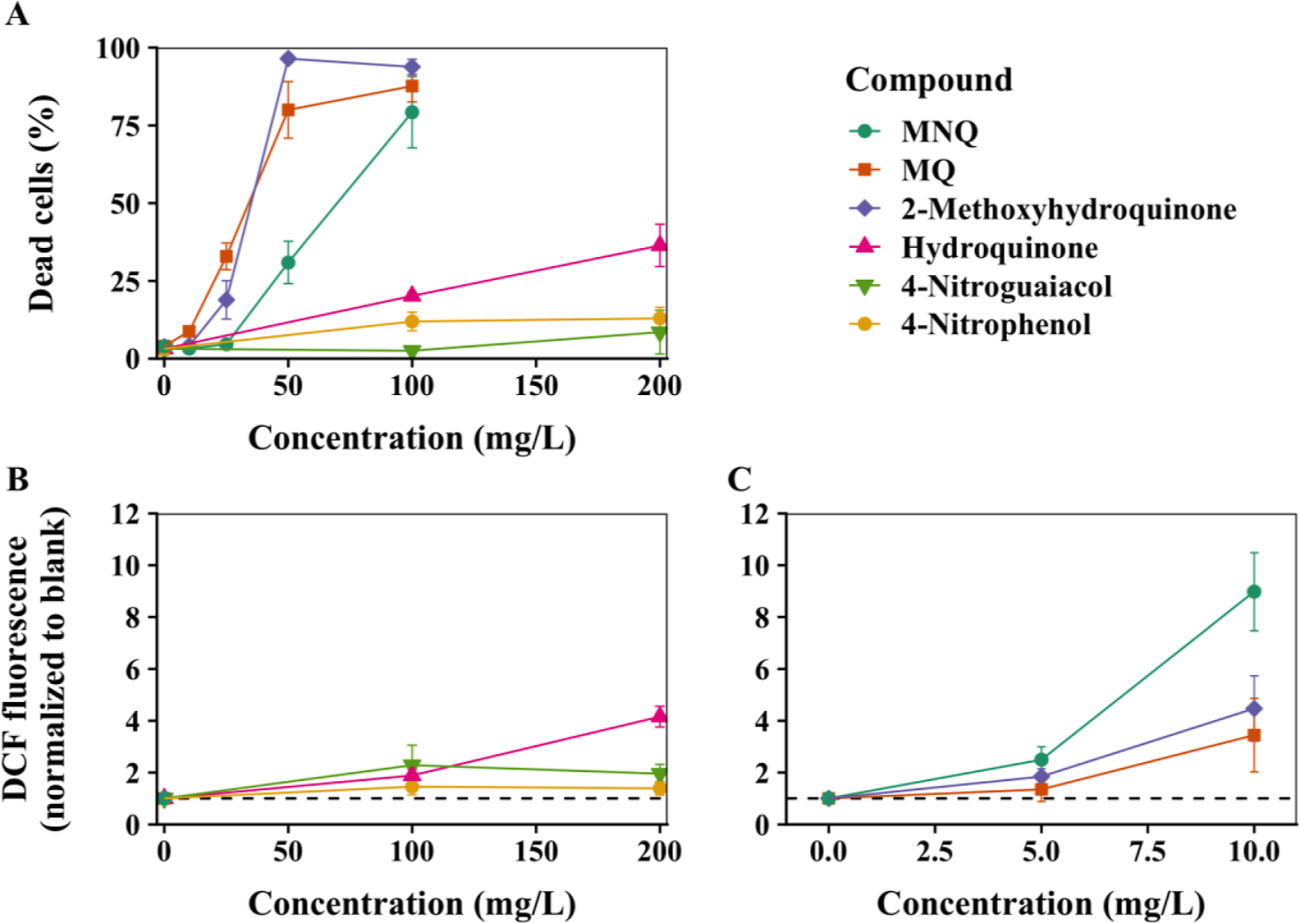
Cell tests of selected chemical standards: (a) cell death after 24 h exposure to all standards; (b) cellular ROS generation after 5 h exposure to 100 and 200 mg L^–1^ hydroquinone, 4-nitrophenol, and 4-nitroguaiacol; and (c) cellular ROS generation after 5 h exposure to 5 and 10 mg L^–1^ 2-methoxyhydroquinone, 2,5-dimethyl-1,4-benzoquinone (MQ), and 2,5-dimethyl-3,6-dinitro-1,4-benzoquinone (MNQ). Points and error bars represent the mean and standard deviation. Raw fluorescence data were normalized to the fluorescence of the corresponding blank from the same experiment; the mean ± standard deviation of normalized values is shown. A dashed line is plotted for reference at *y* = 1. Statistical analyses are summarized in Text S4.

To assess the biological relevance of acellular OP and ROS generation measurements, cellular ROS production and cell death were measured in A549 lung epithelial cells exposed to selected chemical standards and aging conditions of Phc-SOA (Figures 2 and 3). 1,4-Hydroquinone and 4-nitrophenol are representative early-stage products of phenol oxidation in the absence and presence of NO_*x*_, respectively; 2-methoxyhydroquinone and 4-nitroguaiacol represent early products of guaiacol oxidation without and with NO_*x*_, respectively. For each precursor, the compounds representing early oxidation products in the presence of NO_*x*_ were less toxic than those representing oxidation of the same precursor without NO_*x*_: hydroquinone and 2-methoxyhydroquinone induced more cell death and ROS production than 4-nitrophenol and 4-nitroguaiacol, respectively (Figure 3).

Among these, 2-methoxyhydroquinone has the highest cytotoxicity in terms of cellular ROS and cell death. Exposure to 10 mg L^–1^ of 2-methoxyhydroquinone induced a DCF fluorescence increase similar to or greater than the DCF fluorescence of the other three standards at 200 mg L^–1^. Likewise, more than 95% of human lung epithelial cells were dead following exposure to 50 mg L^–1^ of 2-methoxyhydroquinone for 24 h, whereas exposures to hydroquinone and the two phenols were substantially less toxic, causing less than 25% cell death even at 200 mg L^–1^. These results are consistent with our OP measurements and indicate that 2-methoxyhydroquinone and its homologous compounds can be relatively important detrimental products in phenolic SOA.

MQ and MNQ represent non-nitro and nitro-containing quinone compounds, respectively. Quinones may be particularly important components in terms of toxicity: Ito et al.^60^ tested components of *m*-xylene SOA representing various compound classes, including substituted phenols, and found that only the quinone-induced activation of the antioxidant response element (ARE), an oxidative stress response pathway. The two quinones tested here exhibited contrasting trends for ROS and cell death (Figure 3a,c). MNQ generated more cellular ROS than MQ (*p* ≤ 0.005 at all concentrations; Table S4), while exposure to MQ induced higher cell death (*p* ≤ 0.05 at all concentrations; Table S4). The cellular ROS trend was consistent with OP; MNQ had higher OP than MQ, but cell death was not, suggesting that OP can indeed represent the potential for oxidative stress but not necessarily overall toxicity. Other studies comparing the toxicity of different quinones have also observed that trends in cell death do not always correspond with trends in other biological outcomes, including cellular ROS production, intracellular Ca^2+^ levels, and DNA damage.^61,62^ Both cell death and ROS values from MQ and MNQ exposures were higher than hydroquinone and the nitrophenols and comparable to 2-methoxyhydroquinone. Overall, compounds with detectable OP exhibited more cytotoxicity than OP-inert compounds, but the level of cytotoxic effects did not directly correspond to OP values.

### 3.5 In Vitro Toxicity of SOA

**Figure 4:**
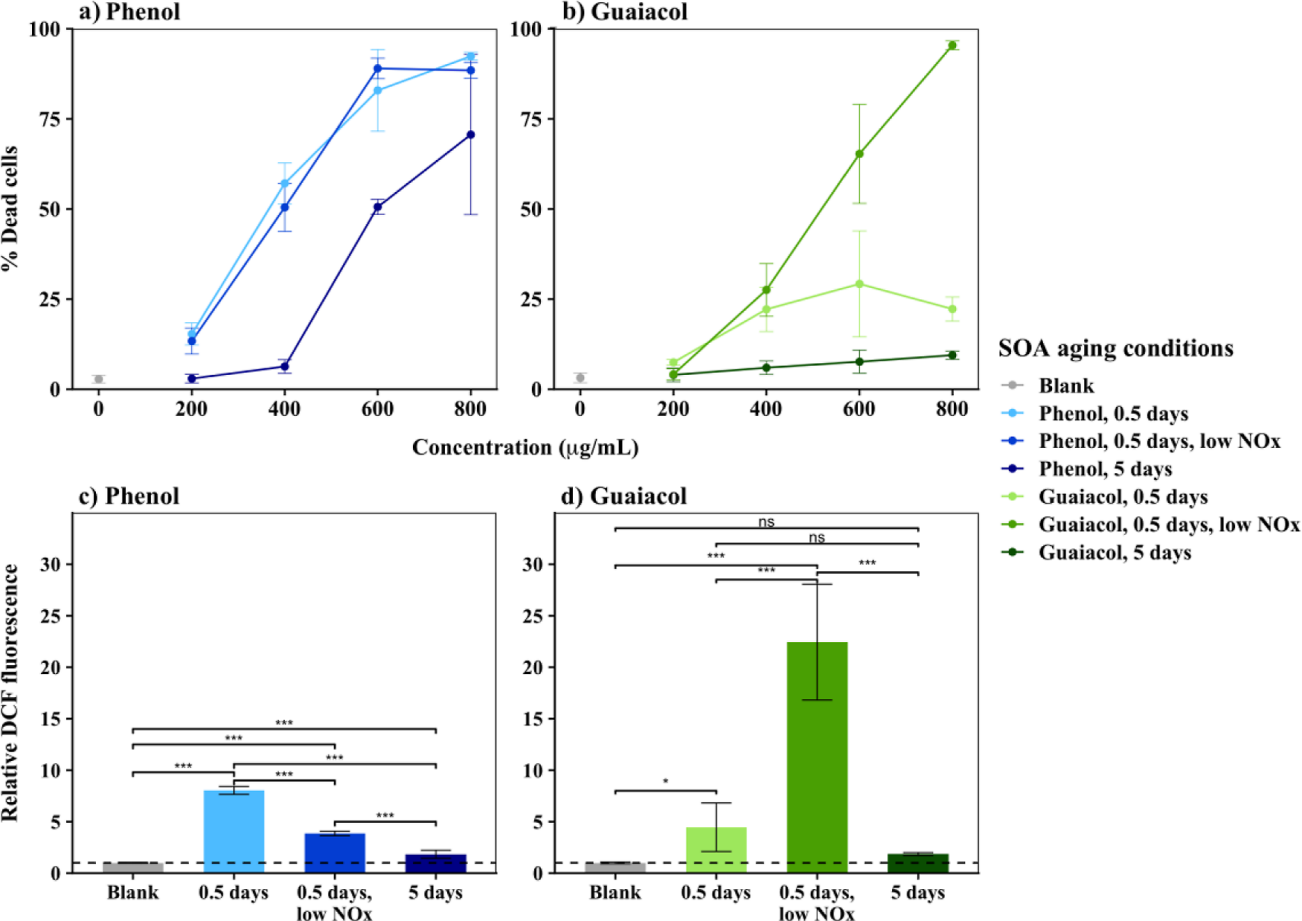
Cell death following 24 h exposure to (a) PSOA and (b) GSOA and cellular ROS generation following 5 h exposure to 400 mg L^–1^ (c) PSOA and (d) GSOA. Points/columns and error bars represent the mean and standard deviation, respectively. Raw DCF fluorescence data were normalized to the fluorescence of the corresponding blank from the same experiment, and a dashed line was plotted for reference at *y* = 1. Average DCF responses were compared between SOA conditions by ANOVA with Tukey HSD to adjust the *p*-values of pairwise comparisons. ***: *p* ≤ 0.005; **: *p* ≤ 0.01; *: *p* ≤ 0.05; ns: *p* > 0.05. Parallel statistical analyses of cell death experiments are summarized in Text S5.

Cell exposure experiments were conducted with both PSOA and GSOA that were generated under the conditions of “0.5 days”, “0.5 days, low NO_*x*_”, and “5 days”. In the measured range of Phc-SOA exposure levels from 200 mg L^–1^ × 24 h to 800 mg L^–1^ × 24 h, cell death increased in a dose-response manner (Figure 4). Our previous study showed that human lung epithelial cells exposed to 300 mg L^–1^ of anisole SOA for 5 h experienced an 8–20% death rate.^38^ Here, 5 h exposures to 400 mg L^–1^ of Phc-SOA resulted in 1–11% cell death (Figure S7), suggesting a higher toxicity of anisole SOA compared to Phc-SOA. Comparing other published exposures of 200 mg L^–1^ × 24 h, the capacity of Phc-SOA to induce human lung epithelial cell death is higher than that of ambient BB aerosols in the Amazon region,^63^ is comparable to SOA generated from ozonolysis of α-pinene,^64^ and is lower than the water-soluble wood tar materials.^65^

By comparing Phc-SOA with photochemical ages of 0.5 days and 5 days, longer photochemical aging decreased the cytotoxicity of both PSOA and GSOA, regardless of NO_*x*_ presence, in terms of both cell death (Figure 4a,b and Table S5; *p* ≤ 0.05 at all concentrations for 5 versus 0.5 days without NO_*x*_ and most concentrations for 5 versus 0.5 days + low NO_*x*_) and cellular ROS measured by the DCF assay (Figure 4c,d). The effect of aging on SOA toxicity varies among the precursors. For example, aging increased the toxicity of naphthalene and α-pinene SOA^45^ but did not change the toxicity of anisole SOA.^38^ Photochemical aging of primary particles can also increase or decrease toxicity, depending on sources and oxidation conditions.^66,67^ In one such study, the aging of smoke from mixed fuels decreased its mutagenicity as well as its PAH content, suggesting that aromaticity could drive toxicity.^68^ Here, when the photochemical age is above 3 days, the carbon yield decreases in PSOA and GSOA (Figure S8), suggesting that fragmentation reactions prevail over functionalization reactions^69^ for both Phc-SOA. From the UHPLC-Orbitrap MS results, we learn that the signal percentage of unambiguous aromatic compounds (aromaticity equivalent ≥2.50) decreased from 23.5% in “0.5 days” PSOA to 12.3% in “5 days” PSOA and decreased from 20.0% in “0.5 days” GSOA to 16.6% in “5 days” GSOA (Table 1). Therefore, open-ring products from fragmentation reactions decreased the cytotoxicity of the Phc-SOA. Decreasing toxicity corresponding with decreased aromaticity is consistent with a recent study: it was found that more-oxygenated organic aerosol (OA) with higher aromaticity, characterized by greater unsaturation and more aromatic ring-retaining components, was the type of OA most strongly associated with cellular ROS production.^46^

The impact of NO_*x*_ on the toxicity differed between GSOA and PSOA and, in the case of PSOA, also between the two measured biological endpoints. For GSOA, NO_*x*_ addition increased the toxicity: “0.5 days, low NO_*x*_” GSOA induced more cell death and higher cellular ROS levels than “0.5 days” GSOA (Figure 4b,d; *p* ≤ 0.005 for cell death at 200, 600, and 800 mg L^–1^ as well as for ROS). In contrast, “0.5 days” PSOA has a larger cellular ROS formation potential than “0.5 days, low NO_*x*_” PSOA (Figure 4c; *p* ≤ 0.005), while the two SOA have comparable cytotoxicity within the explored range of cell exposure levels (Figure 4a). As shown in Table 1, “CHON (O/*N* ≥ 3, %)” is the signal proportion of the assigned nitro compounds and organonitrates in UHPLC-Orbitrap MS,^43^ and adding NO_*x*_ increased the proportion of these compounds. Nitroaromatics, which are known products of phenol/guaiacol oxidation in the presence of NO_*x*_,^7^ can cause dysfunction of the cell membrane and mitochondria and thus threaten cells’ viability.^26,71^ This may explain the comparable death rate between the two types of PSOA when cellular ROS was lower in “0.5 days, low NO_*x*_” PSOA. The signal intensities of 16 possible quinone structures in UHPLC-Orbitrap MS were also explored. As illustrated above, 1,4-benzoquinone and 2-methoxy-1,4-benzoquinone are possible products in PSOA and GSOA under the non-NO_*x*_ condition, respectively.^6,18,34^ Therefore, their parent molecules and OH-adducts C_6_H_4_O_2–6_ and C_7_H_6_O_3–6_ were investigated. With NO_*x*_ involved, we also searched for C_6_H_3_NO_4–7_ and C_7_H_5_O_5–7_, which are quinone products with one nitro group. The structures of the 16 explored quinone species are summarized in Figure S12, and the proportions of their signal intensities are shown in Table S3. Compared with “0.5 days” PSOA, the proportion of nitro-containing quinones in “0.5 days”, low NO_*x*_” PSOA only showed a minimal increase by 0.02%. For “0.5 days” of GSOA, the presence of low NO_*x*_ increased the proportion of nitro-containing quinones by 0.54%. Therefore, the presence of NO_*x*_ led to the addition of a nitro group to quinones in GSOA but not in PSOA. This is consistent with the relatively high cellular ROS signal observed for “0.5 days, low NO_*x*_” GSOA.

## 4 Atmospheric Implications

As a component of accelerating global climate change, wildfire prevalence has been increasing in recent decades, raising broad concerns about the possible health impacts of wildfire smoke. A recent “top-down” global model study reported that wildfire SOA production (139 ± 34 Tg per year) explains at least 30% of the global SOA production,^72^ emphasizing the importance of BB-SOA in the troposphere. BB-SOA usually arises in two main ways: the oxidation of VOCs or the oxidation of semivolatile compounds that evaporate from POA as the plume is heated or diluted. Recent studies highlight the relatively high SOA formation potential of phenolic compounds among known VOCs emitted from BB,^14−16^ while semivolatile compounds in POA are often considered as important SOA precursors that have not been well-identified yet.^15,33,73^ Given this, the fuel-based relative importance of different BB-related VOCs in forming SOA and thus causing OP, denoted as OP_BB-VOC_ (pmol min^–1^ mg^–1^ fuel), is calculated as
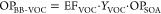
4where EF_VOC_ (g kg^–1^ fuel) is the emission factor of a specific VOC species in BB; *Y*_VOC_ is the SOA yield. As shown in Table S2, OP_phenol_ and OP_guaiacol_ are 3.4–24.9 and 6.8–20.1 pmol min^–1^ mg^–1^ fuel, respectively, which have higher either lower or upper bounds than OP_BB-VOC_ of other explored species, including anisole, naphthalene, 1,3,5-Trimethylbenzene, toluene, *m*-xylene, and biogenic VOCs.^38,51−53,70,74,75^ The secondarily evaporated semivolatile compounds (termed as SBB-OGs therein) from BB-POA were recently shown to generate BB-SOA,^15^ and thus, the OP_SBB-OGs_ (0.4–1.2 pmol min^–1^ mg^–1^ fuel) is an order of magnitude lower than OP_phenol_ and OP_guaiacol_ due to its relatively lower SOA yield and OP_SOA_. Our study shows that Phc-SOA is a significant contributor of the oxidative potential of BB-SOA.

We also identified several ways by which the OP of Phc-SOA is linked to its chemical composition. The toxicity of OH-adducts of guaiacol (e.g., 2-methoxyhydroquinone, 1,4-dihydroxy-2,6-dimethoxybenzene) is confirmed in atmospheric aerosols. An et al. used a theoretical model to predict OH-initiated oxidation products of guaiacol and found that 2-methoxyhydroquinone had the least LC50 values (concentration of tested substance causing 50% of the death rate) for fish and green algae among suggested reaction products.^18^ In our study, the presence of 2-methoxyhydroquinone in GSOA was confirmed by UHPLC-Orbitrap MS. Acellular and cellular assays were used to confirm that the toxicity arises from its high OP, and a potential mechanism was also proposed. The methoxy group promotes the electron-withdrawing ability of polyphenols with *para*-dihydroxy functional groups and thus increases their OP. Methoxy-containing phenolic compounds in BB smoke, such as syringol and eugenol, are likely to generate relatively highly toxic products in fresh BB-SOA. We first reported that the OP of a specific quinone compound increases with the addition of the nitro group, which might explain why the OP of GSOA increased in the presence of NO_*x*_. In vitro measurements on cellular ROS and cell viability both show that the cytotoxicity of “5 days” Phc-SOA is lower than the “0.5 days” Phc-SOA, which is consistent with the decrease in OA aromaticity as its photochemical age grows from 0.5 to 5 days.

By combining different acellular and cellular assays, we can comprehensively understand aerosol toxicological properties. For example, both acellular (OP) and cellular (cell death, DCFH-DA) toxicity of GSOA decreased with aging, showing consistency among very different techniques to estimate aerosol toxicity. In addition to the DTT assay that measures the OP of SOA, EPR spectrometry and the fluorometric H_2_O_2_ assay measured different ROS species that Phc-SOA generates in water and found that H_2_O_2_ is the main ROS species. The comparison between cellular ROS and a cell viability assay following PSOA exposure suggests that oxidative stress is not the only mechanism that accelerates cell death. It is also observed that the ROS yield of GSOA in water followed the order of “5 days” > “0.5 days” ≈ “0.5 days, low NO_*x*_”, while the order was “0.5 days, low NO_*x*_” > “0.5 days” > “5 days” for cellular ROS. Particle-bound and cellular ROS are generally hypothesized to be related, as was observed in a study on naphthalene and α-pinene SOA,^76^ but for various reasons, they may also follow divergent trends. Cellular ROS can comprise particle-bound ROS species and ROS generated by cellular redox reactions in response to PM components.^77^ Additionally, the buffer required for cell exposures is in a different physicochemical environment than pure water (as used for OP measurements), and factors like pH value, temperature, and inorganic ions could influence the ROS formation potential of SOA.^27,78,79^ SOA into cells can also be influenced by its components. For example, nitroaromatics can promote the penetration of SOA components through the cell membrane,^26,71^ which may explain why a higher cellular ROS level was observed for “0.5 days, low NO_*x*_” GSOA compared to the “0.5 days” GSOA without NO_*x*_. Therefore, the acellular and cellular assays measure the ROS without and with biological processes, respectively, and they should be combined to understand the possible biological processes in tissue or the body. The divergence between OP^DTT^ and cellular ROS can be observed in this study and in studies on naphthalene SOA and ambient BB aerosols.^52,80^ We suspect that the phenomenon might be partly caused by antioxidant species (e.g., polyphenols) in Phc-SOA that can relieve cellular ROS but cannot be captured by the DTT assay. We will try to figure out this property in a follow-up study.

## Supporting Information Available

Experimental parameters of SOA formation, procedures of acellular/cellular assays, calibration curves, data analysis, proposed reaction mechanisms, and comparison among different aerosols (PDF)
