# Supplementary material for: Secondary Organic Aerosol Generated from Biomass Burning Emitted Phenolic Compounds: Oxidative Potential, Reactive Oxygen Species, and Cytotoxicity
Source: Environ Sci Technol. 2024 Apr 29;58(19):8194–206. doi: 10.1021/acs.est.3c09903 (PMC11097630; doi:10.1021/acs.est.3c09903)
Supplement: Supplementary file 1 — es3c09903_si_001.pdf [file es3c09903_si_001.pdf]

Supplementary information of

Secondary Organic Aerosol Generated from Biomass Burning Emitted Phenolic Compounds: Oxidative Potential, Reactive Oxygen Species and Cytotoxicity

Zheng Fang [a,*], Alexandra Lai [a], Dongmei Cai [b], Chunlin Li [a,c], Raanan Carmieli [d], Jianmin Chen [b], Xinming Wang [e], Yinon Rudich [a,*]

[a] Department of Earth and Planetary Sciences, Weizmann Institute of Science, Rehovot 76100, Israel

[b] Shanghai Key Laboratory of Atmospheric Particle Pollution and Prevention (LAP 3), Department of Environmental Science and Engineering, Fudan University, Shanghai 200438, China

[c] College of Environmental Science and Engineering, Tongji University, Shanghai 200072, China

[d] Department of Chemical Research Support, Weizmann Institute of Science, Rehovot 76100, Israel

[e] State Key Laboratory of Organic Geochemistry and Guangdong Key Laboratory of Environmental Protection and Resources Utilization, Guangzhou Institute of Geochemistry, Chinese Academy of Sciences, Guangzhou 510640, China

***Correspondence to:**

Zheng Fang (zheng.fang@weizmann.ac.il)

Yinon Rudich (yinon.rudich@weizmann.ac.il)

This supporting information file contains 30 pages with 5 text sections, 5 table, and 12 figures as follows:

- Text S1: Measurements on SOA density and yield
- Text S2: H_2O_2 yield of Phc-SOA in water
- Text S3: Peroxyacyl nitrates (PANs) measurement
- Text S4: Analysis of organic peroxides with the UHPLC-Orbitrap MS
- Text S5: Cell death assay and the DCFH-DA assay
- Table S1: Experimental parameters and abbreviations of Phc-SOA.
- Table S2: The fuel-based relative importance of different BB-related VOCs in forming SOA and thus causing oxidative potential (OP_{VOC}).
- Table S3. Proportions of possible quinone signals in UHPLC-Orbitrap MS.
- Table S4. Statistical analyses of cell death and ROS data presented in Figure 3.
- Table S5. Statistical analyses of SOA cell death data presented in Figure 4.
- Fig. S1: The size-dependent wall loss rate of organic aerosols in the PAM.
- Fig. S2: Calibration curve of Griess assay with water solution of $NaNO_2$.
- Fig. S3: Calibration curves for (a) ROS radicals with EPR analysis, (b) H_2O_2 with fluorescence spectroscopy.
- Fig. S4: OP^{DTT} of BB-related SOA in literature**.**
- Fig. S5: Fragmentation data of 2-methoxyhydroquinone with retention time of 2.47 min in UHPLC-Orbitrap MS.
- Fig. S6: Proposed reaction mechanisms in the manuscript.
- Fig. S7: Cell death following 5h exposure to 400 mg L^{-1} SOA
- Fig. S8: Yields of oxygen and carbon in SOA against photochemical age.

- Fig. S9 Proposed reaction mechanism of how organic peroxides are formed through photochemical aging of phenol and guaiacol.
- Fig. S10 The relationship of DTT consumption rate and the 1,4-NQ concentration.
- Fig. S11 EPR spectra of “0.5 day” guaiacol SOA without and with DMSO.
- Fig. S12 The 16 possible quinone species investigated by UHPLC-Orbitrap MS.

Text S1: Measurements on SOA density and yield

A SMPS and an aerodynamic aerosol classifier (AAC, Cambustion, UK) were used in tandem to measure the effective density of SOA. SOA of a specific aerodynamic diameter was first size selected by the AAC, and then was introduced into the SMPS for size distribution measurement. The size distribution obtained from SMPS was fitted with a lognormal distribution equation to derive its median mobility diameter. By assuming that PhC-SOA is spherical and non-porous, the effective density is calculated as:

$$\rho_{\mathrm{eff}} = \rho_0 \cdot \frac{d_{\mathrm{va}}}{d_{\mathrm{m}}}$$

Eq. 1

where ρ_0 is the standard density (1.0 g cm^{-3}), and d_{va} and d_{m} are the AAC-measured aerodynamic diameter and SMPS-measured mobility diameter, respectively. In each density measurement, SOA of 5 different mobility diameter within the range of 190-350 nm were size-selected. No significant change in effective SOA density was observed among different mobility diameters, and the results for different Phc-SOA are summarized in Table S1. The volume concentration of SOA at the outlet of PAM was measured by SMPS and mass concentration was obtained by multiplying volume concentration and effective density.

The SOA yield is defined as the ratio of SOA mass to the mass of reacted phenolic compounds. Particle loss inside the PAM was characterized by introducing standard polystyrene spheres (PSL, Thermo Fisher) into the PAM, and the size distribution of PSL at inlet and outlet of PAM were measured by SMPS. By treating particle wall loss as a first-order process, the size-dependent wall loss rate of organic aerosols inside the PAM was estimated (Fig. S1). For all types of SOA, the mass concentration for each size bin was

corrected with the wall loss rate and residence time in the PAM before calculating SOA yield.

In the reaction between VOC and OH radical, VOC was consumed at the rate of:

$$\frac{d[VOC]}{dt} = k_{VOC+OH} \cdot [VOC] \cdot [OH] \qquad \text{Eq. 2}$$

where [VOC] and [OH] mean real time concentration of VOC and OH, respectively; t means time, k_{VOC+OH} means the reaction rate constant for this reaction. After doing integration to E1, the following equation can be obtained:

$$\ln\frac{[VOC]_0}{[VOC]_t} = k_{VOC+OH} \cdot \int_0^t [OH]dt \qquad \text{Eq. 3}$$

where $[VOC]_0$ means the initial VOC concentration, $[VOC]_t$ means the VOC concentration at a specific time, and the integrated OH concentration over this period $\int_0^t [OH]dt$ is namely the OH exposure. In each experiment, the $[VOC]_0$ was set to a known value, the OH exposure within the PAM reactor was characterizing by the decay of SO_2, and the k_{VOC+OH} was obtained from literature,[1] so as to acquire $[VOC]_t$ at the outlet of the reactor. The reacted VOC is defined as the difference between $[VOC]_0$ and $[VOC]_t$.

Table S1 summarizes SOA yields for phenol and guaiacol precursors in different aging conditions.

Text S2: H_2O_2 yield of Phc-SOA in water

The Fluorometric Hydrogen Peroxide Assay Kit (MAK165, Sigma) was used to measure the H_2O_2 yield of SOA. Stock solutions of the red peroxidase substrate, horseradish peroxidase, and assay buffer were prepared according to the manufacturer's guidance. Each stock solutions were then separated into aliquots and stored at -20 ^oC. Upon measurement, 950 μL of assay buffer, 10 μL of peroxidase substrate, and 40 μL horseradish peroxidase were mixed to generate the working solution, which must be used within two hours. After

that, 60 μL of the working solution and 2940 μL of the water extract of SOA were then mixed and immediately transferred to a fluorescent plate reader (Biotek, The Synergy HT) for analysis. The excitation wavelength and emission wavelength were 540 nm and 590 nm, respectively, and the fluorescence signals from 25 to 30 min were averaged for the final result. For quantification, the H_2O_2 solutions with concentrations of 0, 0.2, 0.4, 0.6, 0.8, 1.0 μM were made and then measured following the steps above (Fig. S3b). Concentrations of SOA solutions were also carefully diluted before measurements, to generate fluorescence signals lower than the 1.0 μM H_2O_2. To eliminate the interference of organic peroxides in SOA to the H_2O_2 measurement, a control group was set by adding 10 μL of catalase (Sigma-Aldrich, 138 U μL^{-1}) to the SOA extract. We tested the scavenging ability of such a dose of catalase by comparing the "2 μM H_2O_2 + catalase" group and the "pure water + catalase" group, and the two groups proved to generate the same background signals. Because all the tested SOA generated less than 1 μM H_2O_2, the added catalase was enough to fully scavenge H_2O_2. The difference between adding or not adding catalase was used to derive the fluorescence signal induced by H_2O_2. All H_2O_2 measurements were conducted in duplicate.

Text S3: Peroxyacyl nitrates (PANs) measurement

Total PANs were measured by first transforming them into nitrites with alkaline solution, and then convert the nitrites to azo dyes with the Griess reagent.[2,3] SOA were first extracted with pure water and filtered (SLLGC13NL, Millex-LG), then 425 μL of SOA solution was immediately mixed with 425 μL KOH solution (125 mM) for 10 min, during which time PANs were converted to nitrites. After that, 850 μL Griess reagent (aqueous solution of 50 mM sulfanilic acid, 12.5 mM *n*-(1-naphthyl)ethylenediamine dihydrochloride), and 400

mM acetic acid) was added to the mixed solution and react for 30 min to turn nitrites into azo dyes. Absorbance of the solution was measured at 491 nm by UV-Vis spectroscopy to quantify the amount of formed azo dyes. To correct for background absorbance by SOA components, a parallel sample was measured without *n*-(1-naphthyl)ethylenediamine dihydrochloride in the Griess reagent. $NaNO_2$ solutions with concentration ranging from 0 to 30 μM were used to derive a calibration curve for the Griess assay (Fig. S2). Based on the assumptions that: (1) one mole of PAN is transferred to one mole of nitrite, and (2) SOA has an average molecular weight of 200, the proportion of PANs in SOA can be estimated. It is noted that for Phc-SOA produced without NO_x, the proportion of PANs was not zero (e.g., 0.7% in "5 days" PSOA), which could be attributable to impurities present in the N_2. Therefore, the NO_x-involved SOA were further background-corrected with their counterpart non-NO_x SOA.

Text S4: Analysis of organic peroxides with the UHPLC-Orbitrap MS

The possible organic peroxides formed in Phc-SOA were inferred with reaction pathways (Fig. S9) summarized in previous studies.[4] The OH-adducts of the products in reaction 8-11 are also organic peroxides. In this way, the possible organic peroxides $C_6H_8O_{4\text{-}8}$ in PSOA and $C_7H_{10}O_{5\text{-}9}$ in GSOA, respectively. Besides, the methoxy group in guaiacol can be replaced by hydroxy group through OH oxidation, resulting in dihydroxy benzene.[4] Therefore, $C_6H_8O_{5\text{-}8}$ are also possible organic peroxides in GSOA. Since none of those chemicals are commercially available to date, we tentatively assigned signals from all these molecules as organic peroxides, which served as upper-limit estimates. Only molecular ions were taken into count, not their fragment ions.

Text S5: Cell death assay and the DCFH-DA assay

Cell death was measured using a propidium iodide (PI), a membrane-impermeable DNA-intercalating dye that is excluded by viable cells. Following 24-hour exposures to samples (SOA extracts or chemical standard solutions), cells were dissociated with trypsin, resuspended in phosphate-buffered saline (PBS) with PI, and incubated at room temperature in the dark for 15 minutes. Fluorescence was measured by flow cytometry (Amnis CellStream, Luminex, USA) with excitation and emission wavelengths of 488 and 610 nm, respectively.

Cellular ROS were measured using 2',7'-dichlorofluorescein diacetate (DCFH-DA), a fluorescent probe that reacts with a wide range of ROS.[5] Cells were washed with PBS and incubated with 10 μM DCFH-DA in the dark at 37˚C for 30 minutes. The probe solution was removed, cells were washed twice more with PBS, and sample solutions in SGM were added. Exposures for the ROS assay were shorter than for the cell death assay (5 hours instead of 24) due to the transient nature of ROS and cellular processes that produce ROS. After 5 hours of exposure, samples were removed, cells were detached using trypsin, and resuspended in PBS. Fluorescence was measured by flow cytometry with excitation and emission wavelengths of 488 and 529 nm, respectively. The sample fluorescence was blank-normalized using a blank from the same plate as the sample.

Table S1 Experimental parameters and abbreviations of Phc-SOA.

VOC	Photochemical age (day)	$(RO_2+NO)/(RO_2+HO_2)$	Abbreviation	Reacted VOC concentration (ppbv)	SOA yield
Phenol	0.5	0	"0.5 days" PSOA	879±44	0.38±0.04
Phenol	0.5	0.4	"0.5 days, low NO_x" PSOA	2276±114	0.43±0.05
Phenol	5	0	"5 days" PSOA	1030±52	0.79±0.09
Phenol	5	0.4	"5 days, low NO_x" PSOA	909±45	0.31±0.03
Phenol	5	1.0	"5 days, high NO_x" PSOA	672±34	0.27±0.03
Guaiacol	0.5	0	"0.5 days" GSOA	557±28	0.51±0.06
Guaiacol	0.5	0.4	"0.5 days, low NO_x" GSOA	1002±50	0.88±0.10
Guaiacol	5	0	"5 days" GSOA	561±28	0.56±0.06
Guaiacol	5	0.4	"5 days, low NO_x" GSOA	404±20	0.41±0.05
Guaiacol	5	1.0	"5 days, high NO_x" GSOA	250±13	0.39±0.04

Table S2. The fuel-based relative importance of different BB-related VOCs in forming SOA and thus causing oxidative potential (OP_{VOC}, pmol min^{-1} mg^{-1} fuel).

VOC	EF (g kg^{-1} fuel)[a]	SOA yield[b]	OP_{SOA}[c]	Reference for OP_{SOA}	OP_{VOC}[d]
Phenol	0.55	0.13-0.54	48.9-83.8	This study	3.4-24.9
Guaiacol	0.48	0.35-0.50	39.8-83.9	This study	6.8-20.1
Anisole	0.46	0.27-0.49	22.2-73.9	Li et al.[6]	2.8-16.7
Naphthalene	0.07	0.36-0.74	106-259	McWhinney et al;[7] Tuet et al;[8] Zhang et al.[9]	2.7-13.4
135-TMB	0.056	0.04-0.40	14	Jiang et al.[10]	0.0-0.3
Toluene	0.24	0.08-0.66	36.5-68.9	Jiang et al.[11]	0.7-10.9
m-xylene	0.13	0.04-0.40	16-43	Tuet et al.[12]	0.1-2.2
Isoprene	0.21	0.01-0.06	10-12	Tuet et al.[12]	0.0-0.2
Monoterpenes	1.1	0.00-0.33	20-80	Tuet et al;[12] Zhang et al.[9]	0.0-29.0
SBB-OGs	1.2-2.6	0.02-0.03	15.5	Fang et al.[13]	0.4-1.2

[a] All data except SBB-OGs (secondarily evaporated BB organic gases) are from Koss et al.[14] The primary organic aerosols (POA) from BB contain a significant fraction of semivolatile organic compounds, and can release them into the gas phase during the dilution process in transport. Such evaporated compounds were termed as SBB-OGs. Fang et al.[13] reported 2.99-5.96 g kg^{-1} fuel for EFs of BB-POA at ambient-level dilution ratios (1300-4000), and May et al.[15] reported that 40-43% mass of BB-POA evaporated when isothermally (at 9 °C or 25 °C) diluted from an typical ambient (50 μg m^{-3}) to an ultimately diluted concentration (1 μg m^{-3}). By combining the primary emission and the dilution process, we estimate that the EF of SBB-OGs is in the range of 1.2-2.6 g kg^{-1} fuel.

[b] Data of phenol to anisole, guaiacol, naphthalene, 135-TMB, toluene, *m*-xylene and anisole are from Fang et al.,[16] with the SOA yield of 135-TMB assumed to be the same with xylene, and the SOA yield of anisole assumed to be the same with cresol. Isoprene data is from Lamkaddam et al.,[17] assuming OA mass concentration to be 10-1000 μg m^{-3}. Monoterpene data is from Friedman et al.[18] and Liu et al.[19] Data of SBB-OGs is from Fang et al.

[c] In unit of pmol min^{-1} μg^{-1}.

[d] In unit of pmol min^{-1} mg^{-1} fuel.

Table S3. Proportions of possible quinone signals in UHPLC-Orbitrap MS spectra. In total, 16 quinone species were studies, and they were further separated into 7 nitro-containing species and 9 non-nitro species. Note that their isomers in Phc-SOA could have positive biases. On the other side, specific quinone compounds might not be fully dissolved in water, which had negative biases.

VOC	Age (day)	$(RO_2+NO)/(RO_2+HO_2)$	Nitro-containing quinones (%)	Non-nitro quinones (%)	Total quinones (%)
Phenol	0.5	0	0.00	1.36	1.36
Phenol	0.5	0.4	0.02	1.61	1.63
Phenol	5	0	0.00	2.09	2.09
Phenol	5	1.0	0.04	2.35	2.39
Guaiacol	0.5	0	0.00	4.36	4.36
Guaiacol	0.5	0.4	0.54	3.60	4.13
Guaiacol	5	0	0.00	2.81	2.81
Guaiacol	5	1.0	0.32	2.07	2.39

Table S4. Statistical analyses of cell death and ROS data presented in Figure 3[a].

**Cell death (% dead cells, measured with PI)**		
**Concentration (µg/mL)**	**Pairwise comparison**	**Adjusted *p*-value significance[b]**
10	MNQ - MHQ	ns
	MQ - MHQ	***
	MQ - MNQ	***
25	MNQ - MHQ	***
	MQ - MHQ	***
	MQ - MNQ	***
50	MNQ - MHQ	***
	MQ - MHQ	***
	MQ - MNQ	***
100	4NP - 4NG	***
	HQ - 4NG	ns
	HQ - 4NP	***
	MHQ - 4NG	***
	MHQ - 4NP	***
	MHQ - HQ	***
	MNQ - 4NG	***
	MNQ - 4NP	ns
	MNQ - HQ	ns
	MNQ - MHQ	***
	MQ - 4NG	***
	MQ - 4NP	***
	MQ - HQ	***
	MQ - MHQ	***
	MQ - MNQ	*
200	4NP - 4NG	***
	HQ - 4NG	ns
	HQ - 4NP	***
**Cellular ROS (blank-normalized DCF fluorescence)**		
**Concentration (µg/mL)**	**Pairwise comparison**	**Adjusted *p*-value significance[b]**
5	MNQ - MHQ	*
	MQ - MHQ	ns
	MQ - MNQ	***
10	MNQ - MHQ	***
	MQ - MHQ	ns
	MQ - MNQ	***
100	4NP - 4NG	*
	HQ - 4NG	ns
	HQ - 4NP	ns
200	4NP - 4NG	**
	HQ - 4NG	***
	HQ - 4NP	***

[a] Cell death and ROS data were each compared between standards within each tested concentration. 4NP: 4-nitrophenol; 4NG: 4-nitroguaiacol; HQ: hydroquinone; MHQ: 2-methoxyhydroquinone; MNQ: 2,5-dimethyl-3,6-dinitro-1,4-benzoquinone; MQ: 2,5-dimethyl-1,4-benzoquinone.

[b] *P*-values were adjusted using the Tukey HSD test. ***: $p \leq 0.005$; **: $p \leq 0.01$; *: $p \leq 0.05$; ns: $p > 0.05$.

Table S5. Statistical analyses of SOA cell death data presented in Figure 4[a].

Type of SOA	Concentration (µg/mL)	Pairwise comparison	Adjusted *p*-value significance[b]
		0.5 days, low NO_x-0.5 days	***
GSOA	200 µg/mL	5 days-0.5 days	***
		5 days-0.5 days, low NO_x	ns
		0.5 days, low NO_x-0.5 days	ns
GSOA	400 µg/mL	5 days-0.5 days	***
		5 days-0.5 days, low NO_x	***
		0.5 days, low NO_x-0.5 days	***
GSOA	600 µg/mL	5 days-0.5 days	*
		5 days-0.5 days, low NO_x	***
		0.5 days, low NO_x-0.5 days	***
GSOA	800 µg/mL	5 days-0.5 days	***
		5 days-0.5 days, low NO_x	***
		0.5 days, low NO_x-0.5 days	ns
PSOA	200 µg/mL	5 days-0.5 days	***
		5 days-0.5 days, low NO_x	***
		0.5 days, low NO_x-0.5 days	*
PSOA	400 µg/mL	5 days-0.5 days	***
		5 days-0.5 days, low NO_x	***
		0.5 days, low NO_x-0.5 days	ns
PSOA	600 µg/mL	5 days-0.5 days	***
		5 days-0.5 days, low NO_x	***
		0.5 days, low NO_x-0.5 days	ns
PSOA	800 µg/mL	5 days-0.5 days	*
		5 days-0.5 days, low NO_x	ns

[a] In order to compare trends between different SOA conditions, cell death data was compared within each type of SOA and concentration tested

[b] *P*-values were adjusted using the Tukey HSD test. ***: $p \leq 0.005$; **: $p \leq 0.01$; *: $p \leq 0.05$; ns: $p > 0.05$

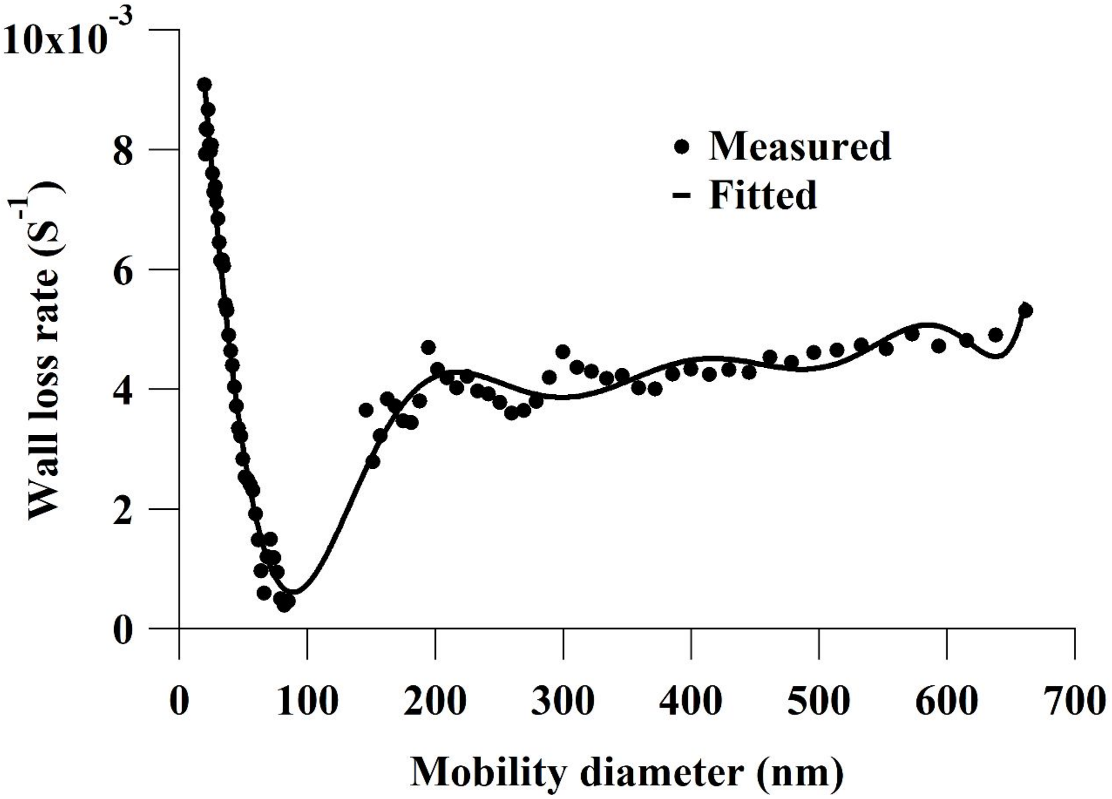

Fig. S1. The size-dependent wall loss rate of organic aerosols in the PAM. For aerosols within specific size bins, particle wall loss rates were not available because of low concentration. To derive those missing wall loss rates, a polynomial regression was applied to fit the relation of wall loss rate and particle size.

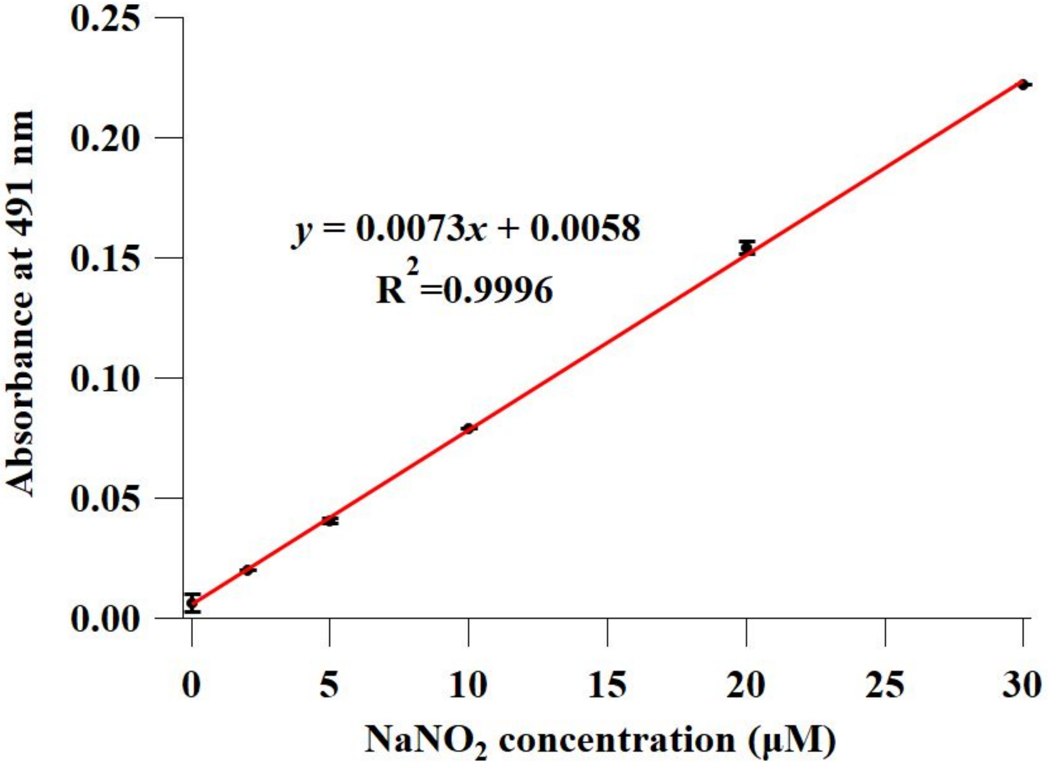

Fig. S2 Calibration curve of Griess assay with water solution of $NaNO_2$. Error bar means the standard deviation derived from two measurements.

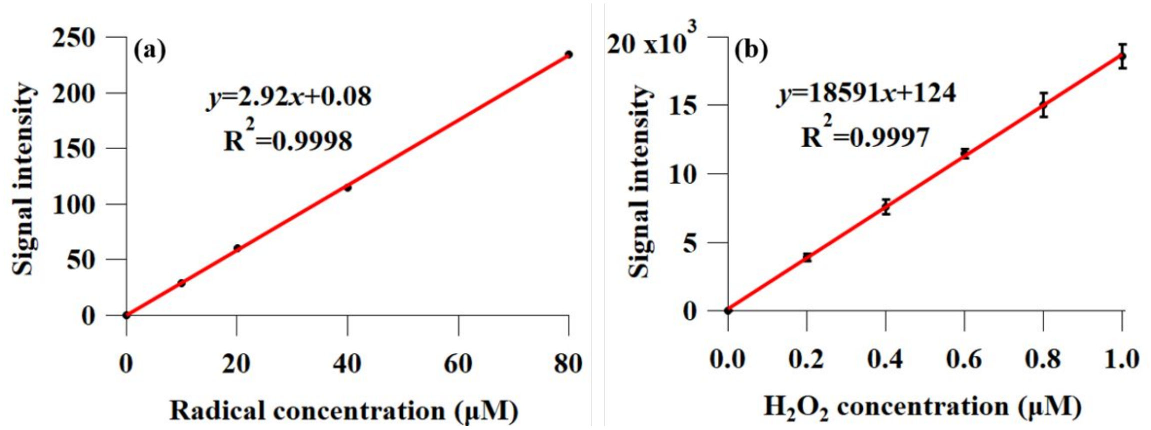

Fig. S3 Calibration curves for (a) ROS radicals with EPR analysis, (b) H_2O_2 with fluorescence spectroscopy. As a stable nitroxide radical, 3-carboxy-proxyl was used to derive the calibration curve for EPR analysis.

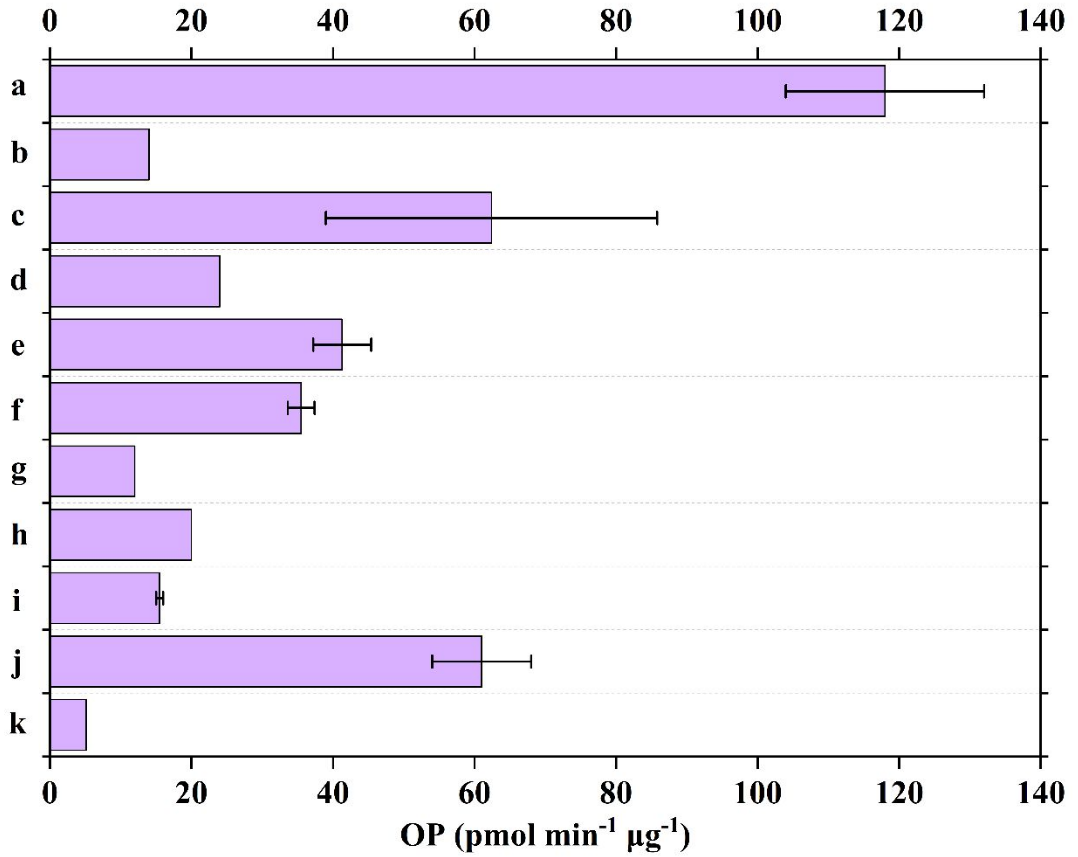

Fig. S4 OP^{DTT} of BB-related SOA in literature. a-i are SOA generated from BB-related VOCs in chamber studies; j-k are BB aerosols measured in ambient air. a: Naphthalene SOA generated with low NO_x.[7] b: Trimethylbenzene SOA, with low NO_x.[10] c: Toluene SOA, with low NO_x.[3] d: *m*-Xylene SOA, with low NO_x.[12] e: Anisole SOA, with low NO_x.[6] f: Anisole SOA, with high NO_x.[6] g: Isoprene SOA, with low NO_x.[12] h: α-Pinene SOA, with low NO_x.[12] i: SOA from secondarily evaporated BB organic gases, without NO_x.[13] j: Ambient BB aerosols.[20] k: Ambient BB aerosols, an OM/OC ratio of 2 is assumed.[21]

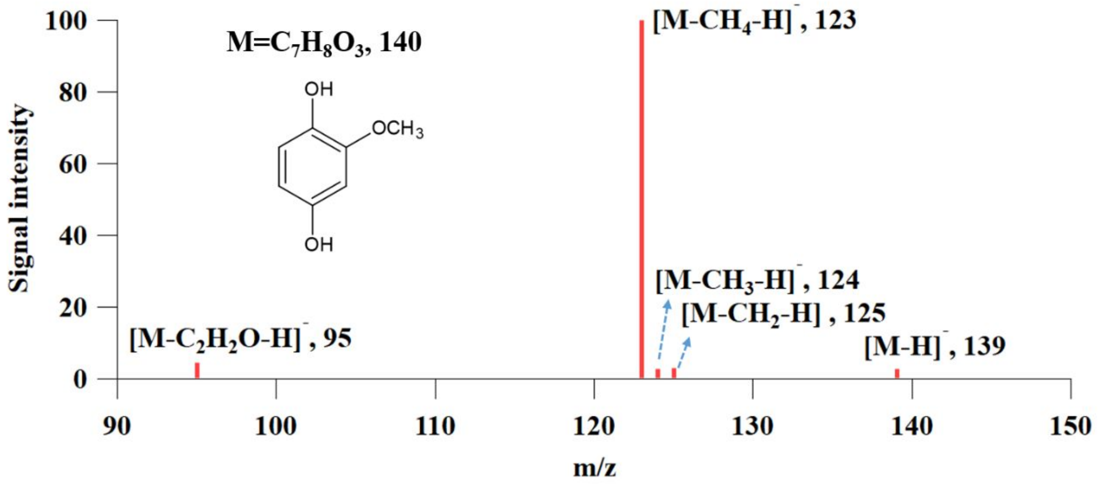

Fig. S5 Fragmentation data of 2-methoxyhydroquinone with retention time of 2.47 min in UHPLC-Orbitrap MS. All signals are normalized by $C_6H_3O_3^-$ at m/z 123.0088, which is the most abundant fragmentation ion.

(a) (b) (c)

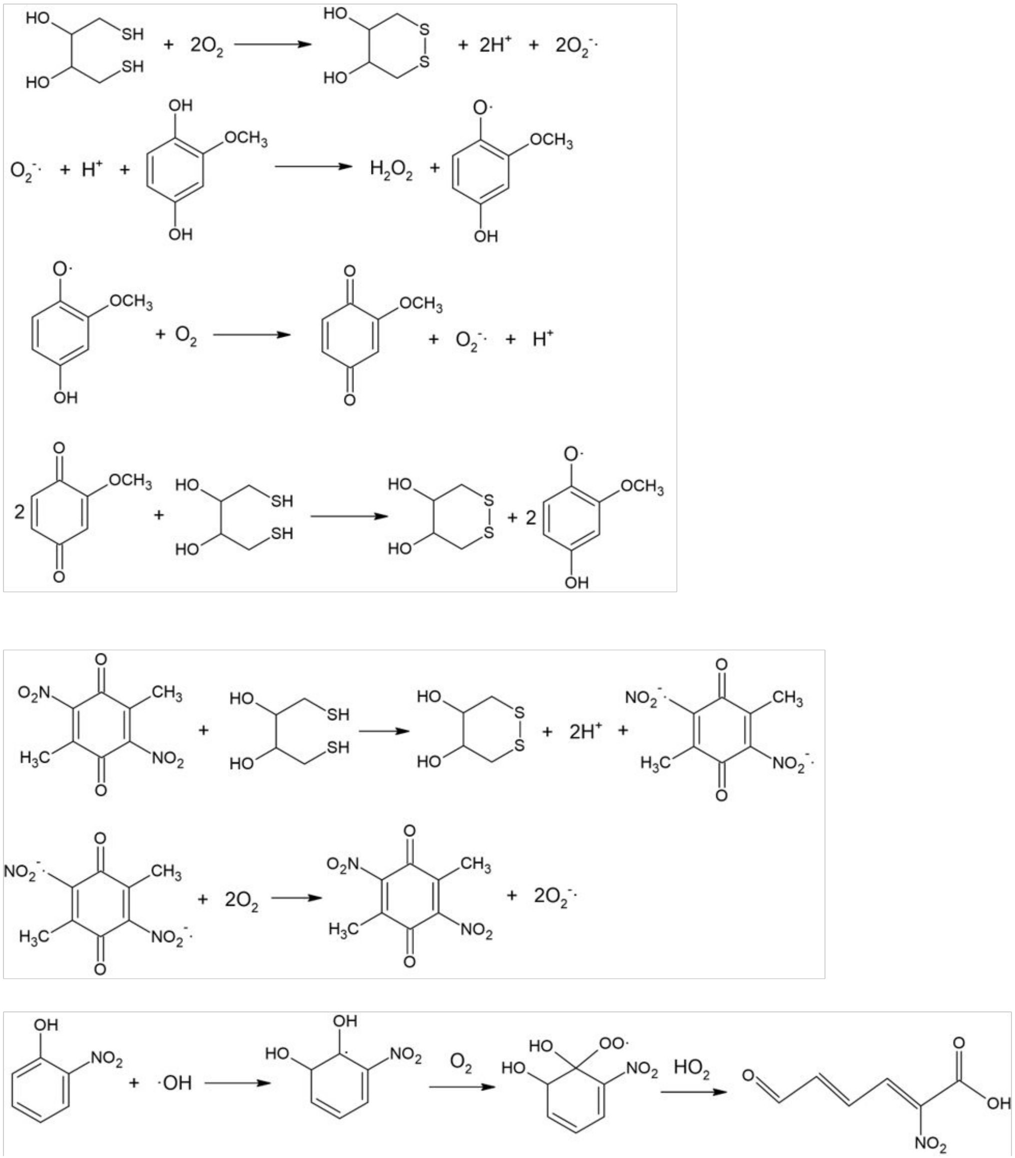

(1) (2) (3) (4) (5) (6) (7)

Fig. S6 Proposed reaction mechanisms for: (a) methoxyhydroquinone catalyzes the consumption of DTT; (b) 2,5-dimethyl-3,6-ditro-1,4-benzoquinone catalyzes the consumption of DTT; (c) nitro-containing aromatics open benzene ring and generate electron-deficient alkenes.

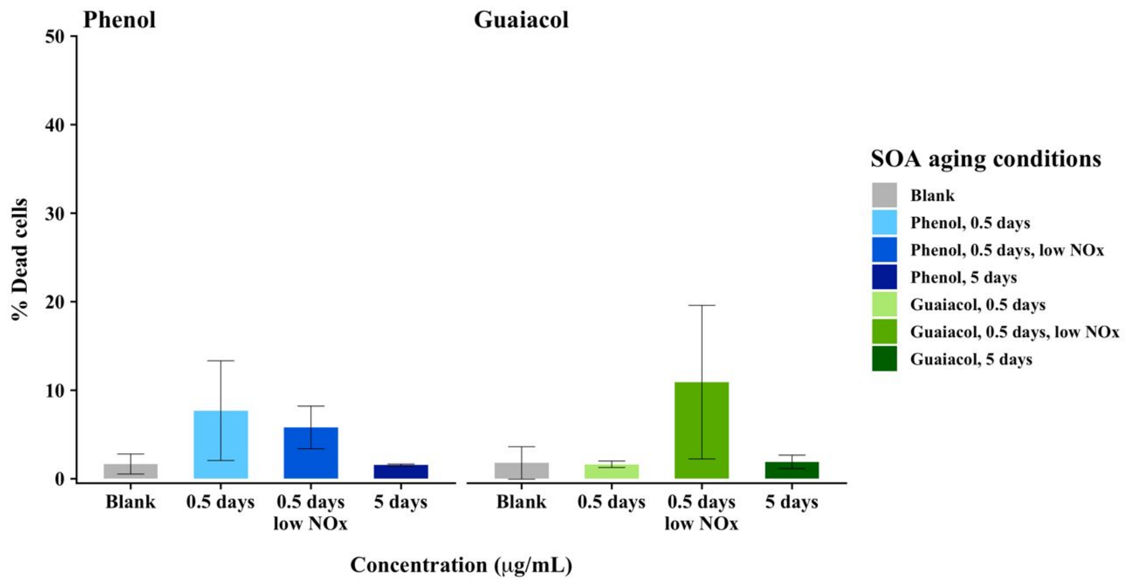

Fig. S7 Cell death following 5h exposure to 400 mg L^{-1} SOA

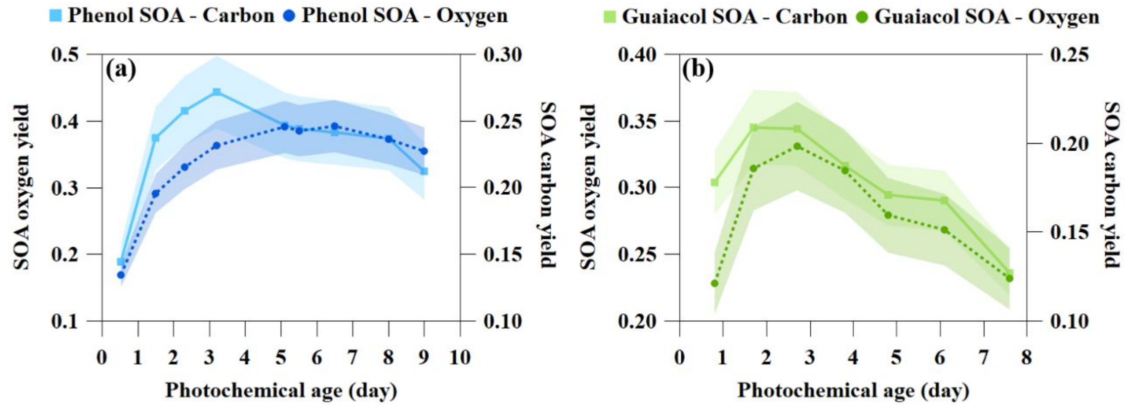

Fig. S8 Yields of oxygen and carbon in SOA against photochemical age for (a) phenol SOA and (b) guaiacol SOA. Errors are depicted as shades.

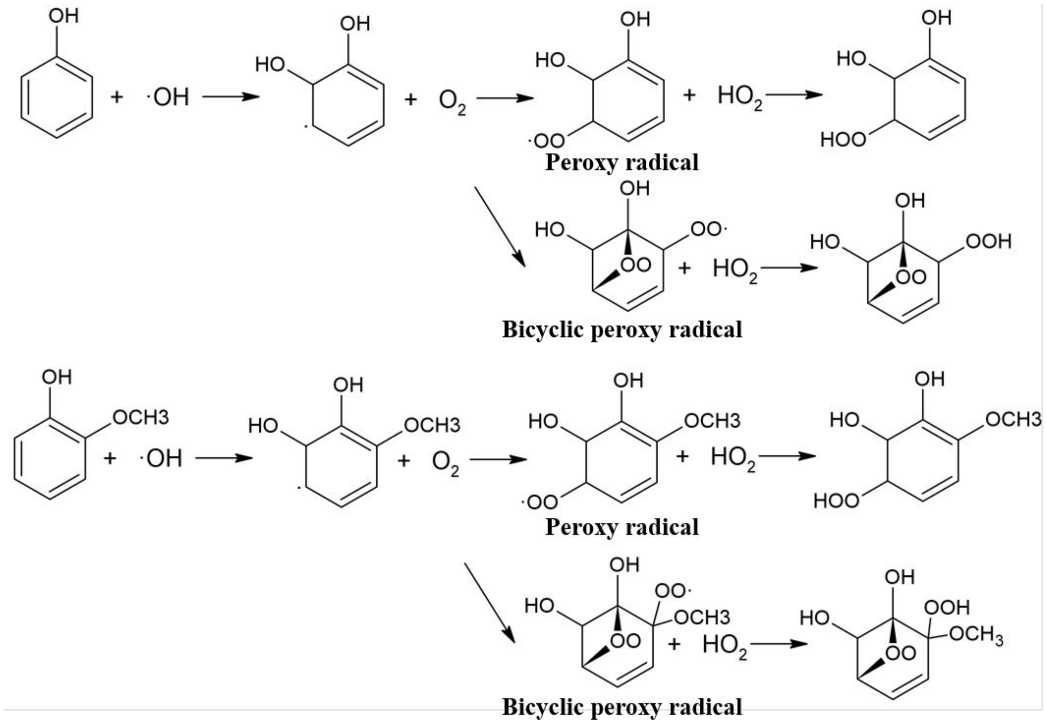

(8)

(9)

(10)

(11)

Fig. S9 Proposed reaction mechanism of how organic peroxides are formed through photochemical aging of phenol and guaiacol.

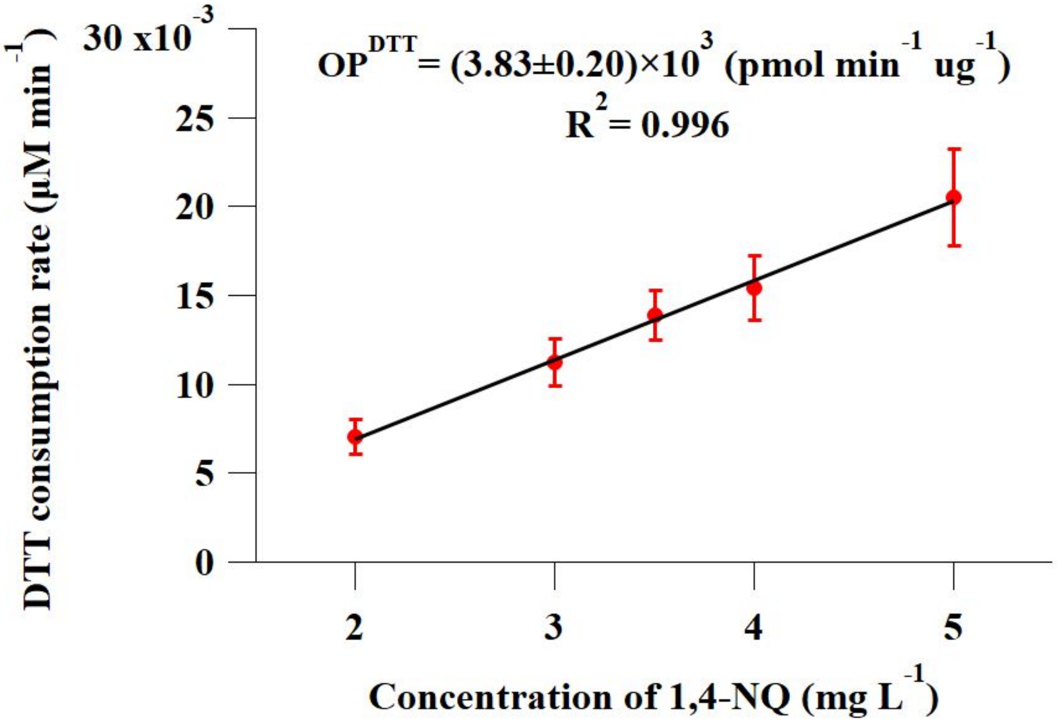

Fig. S10 The relationship of DTT consumption rate and the 1,4-NQ concentration. 1,4-NQ was used as a positive control.

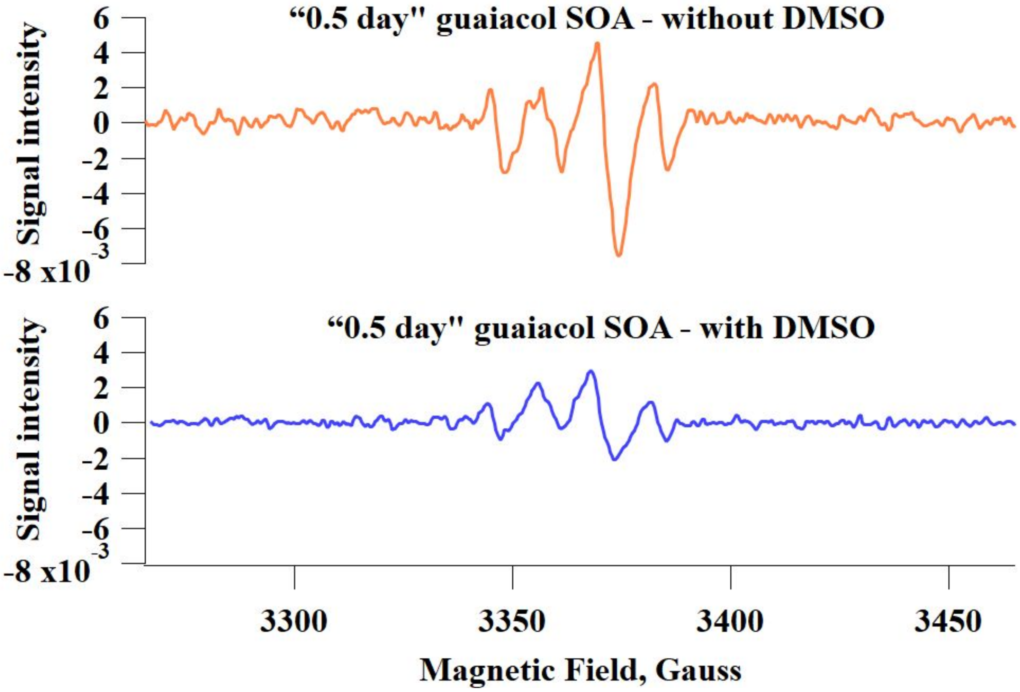

257 **Fig. S11** The EPR spectra of “0.5 day” guaiacol SOA without DMSO (orange line) and

258 with DMSO (blue).

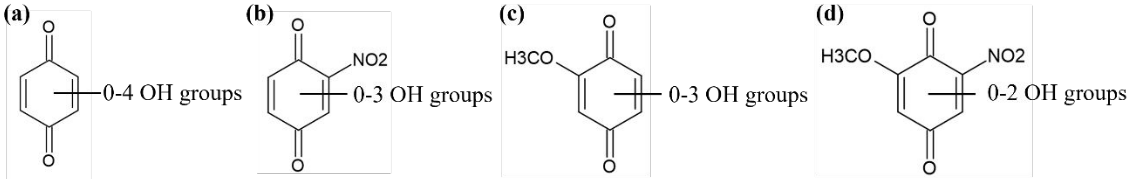

Fig. S12 The 16 possible quinone species investigated by UHPLC-Orbitrap MS. (a) 1,4-Benzoquinones with 0-4 additional hydroxy groups; (b) 2-Nitro-1,4-benzoquinones with 0-3 additional hydroxy groups; (c) 2-Methoxy-1,4-benzoquinones with 0-3 additional hydroxy groups; (d) 2-Methoxy-6-nitro-1,4-benzoquinones with 0-2 additional hydroxy groups.

References

(1) Lewis, S. W.; Agg, K. M.; Gutowski, S. J.; Ross, P. FORENSIC SCIENCES | Gunshot Residues; Worsfold, P., Townshend, A., Poole, C. B. T.-E. of A. S. (Second E., Eds.; Elsevier: Oxford, 2005; pp 430–436. https://doi.org/10.1016/B0-12-369397-7/00204-1.

(2) Jiang, H.; Jang, M.; Yu, Z. Dithiothreitol Activity by Particulate Oxidizers of SOA Produced from Photooxidation of Hydrocarbons under Varied NO_x Levels. Atmos. Chem. Phys. 2017, 17 (16), 9965–9977. https://doi.org/10.5194/acp-17-9965-2017.

(3) Yee, L. D.; Kautzman, K. E.; Loza, C. L.; Schilling, K. A.; Coggon, M. M.; Chhabra, P. S.; Chan, M. N.; Chan, A. W. H.; Hersey, S. P.; Crounse, J. D.; Wennberg, P. O.; Flagan, R. C.; Seinfeld, J. H. Secondary Organic Aerosol Formation from Biomass Burning Intermediates: Phenol and Methoxyphenols. Atmos. Chem. Phys. 2013, 13 (16), 8019–8043. https://doi.org/10.5194/acp-13-8019-2013.

(4) Li, J.; Li, J.; Wang, G.; Ho, K. F.; Dai, W.; Zhang, T.; Wang, Q.; Wu, C.; Li, L.; Li, L.; Zhang, Q. Effects of Atmospheric Aging Processes on in Vitro Induced Oxidative Stress and Chemical Composition of Biomass Burning Aerosols. J. Hazard. Mater. 2021, 401, 123750. https://doi.org/10.1016/j.jhazmat.2020.123750.

(5) Li, C.; Misovich, M. V; Pardo, M.; Fang, Z.; Laskin, A.; Chen, J.; Rudich, Y. Secondary Organic Aerosol Formation from Atmospheric Reactions of Anisole and Associated Health Effects. Chemosphere 2022, 136421. https://doi.org/10.1016/j.chemosphere.2022.136421.

(6) McWhinney, R. D.; Zhou, S.; Abbatt, J. P. D. Naphthalene SOA: Redox Activity and Naphthoquinone Gas–Particle Partitioning. Atmos. Chem. Phys. 2013, 13 (19),

9731–9744. https://doi.org/10.5194/acp-13-9731-2013.

(7) Tuet, W. Y.; Chen, Y.; Fok, S.; Gao, D.; Weber, R. J.; Champion, J. A.; Ng, N. L. Chemical and Cellular Oxidant Production Induced by Naphthalene Secondary Organic Aerosol (SOA): Effect of Redox-Active Metals and Photochemical Aging. Sci. Rep. 2017, 7 (1), 15157. https://doi.org/10.1038/s41598-017-15071-8.

(8) Zhang, Z.-H.; Hartner, E.; Utinger, B.; Gfeller, B.; Paul, A.; Sklorz, M.; Czech, H.; Yang, B. X.; Su, X. Y.; Jakobi, G.; Orasche, J.; Schnelle-Kreis, J.; Jeong, S.; Gröger, T.; Pardo, M.; Hohaus, T.; Adam, T.; Kiendler-Scharr, A.; Rudich, Y.; Zimmermann, R.; Kalberer, M. Are Reactive Oxygen Species (ROS) a Suitable Metric to Predict Toxicity of Carbonaceous Aerosol Particles? Atmos. Chem. Phys. 2022, 22 (3), 1793–1809. https://doi.org/10.5194/acp-22-1793-2022.

(9) Jiang, H.; Jang, M.; Sabo-Attwood, T.; Robinson, S. E. Oxidative Potential of Secondary Organic Aerosols Produced from Photooxidation of Different Hydrocarbons Using Outdoor Chamber under Ambient Sunlight. Atmos. Environ. 2016, 131, 382–389. https://doi.org/10.1016/j.atmosenv.2016.02.016.

(10) Jiang, H.; Jang, M.; Yu, Z. Dithiothreitol Activity by Particulate Oxidizers of SOA Produced from Photooxidation of Hydrocarbons under Varied NO_x Levels. Atmos. Chem. Phys. 2017, 17 (16), 9965–9977. https://doi.org/10.5194/acp-17-9965-2017.

(11) Tuet, W. Y.; Chen, Y.; Fok, S.; Champion, J. A.; Ng, N. L. Inflammatory Responses to Secondary Organic Aerosols (SOA) Generated from Biogenic and Anthropogenic Precursors. Atmos. Chem. Phys. 2017, 17 (18), 11423–11440. https://doi.org/10.5194/acp-17-11423-2017.

(12) Koss, A. R.; Sekimoto, K.; Gilman, J. B.; Selimovic, V.; Coggon, M. M.; Zarzana,

K. J.; Yuan, B.; Lerner, B. M.; Brown, S. S.; Jimenez, J. L.; Krechmer, J.; Roberts, J. M.; Warneke, C.; Yokelson, R. J.; de Gouw, J. Non-Methane Organic Gas Emissions from Biomass Burning: Identification, Quantification, and Emission Factors from PTR-ToF during the FIREX 2016 Laboratory Experiment. Atmos. Chem. Phys. 2018, 18 (5), 3299–3319. https://doi.org/10.5194/acp-18-3299-2018.

(13) Fang, Z.; Deng, W.; Zhang, Y.; Ding, X.; Tang, M.; Liu, T.; Hu, Q.; Zhu, M.; Wang, Z.; Yang, W.; Huang, Z.; Song, W.; Bi, X.; Chen, J.; Sun, Y.; George, C.; Wang, X. Open Burning of Rice, Corn and Wheat Straws: Primary Emissions, Photochemical Aging, and Secondary Organic Aerosol Formation. Atmos. Chem. Phys. 2017, 17 (24), 14821–14839. https://doi.org/10.5194/acp-17-14821-2017.

(14) Lamkaddam, H.; Dommen, J.; Ranjithkumar, A.; Gordon, H.; Wehrle, G.; Krechmer, J.; Majluf, F.; Salionov, D.; Schmale, J.; Bjelić, S.; Carslaw, K. S.; Haddad, I. El; Baltensperger, U. Large Contribution to Secondary Organic Aerosol from Isoprene Cloud Chemistry. Sci. Adv. 2021, 7 (13), eabe2952. https://doi.org/10.1126/sciadv.abe2952.

(15) Friedman, B.; Farmer, D. K. SOA and Gas Phase Organic Acid Yields from the Sequential Photooxidation of Seven Monoterpenes. Atmos. Environ. 2018, 187, 335–345. https://doi.org/10.1016/j.atmosenv.2018.06.003.

(16) Liu, J.; D'Ambro, E. L.; Lee, B. H.; Schobesberger, S.; Bell, D. M.; Zaveri, R. A.; Zelenyuk, A.; Thornton, J. A.; Shilling, J. E. Monoterpene Photooxidation in a Continuous-Flow Chamber: SOA Yields and Impacts of Oxidants, NO_x, and VOC Precursors. Environ. Sci. Technol. 2022, 56 (17), 12066–12076. https://doi.org/10.1021/acs.est.2c02630.

(17) Fang, Z.; Li, C.; He, Q.; Czech, H.; Gröger, T.; Zeng, J.; Fang, H.; Xiao, S.; Pardo, M.; Hartner, E.; Meidan, D.; Wang, X.; Zimmermann, R.; Laskin, A.; Rudich, Y. Secondary Organic Aerosols Produced from Photochemical Oxidation of Secondarily Evaporated Biomass Burning Organic Gases: Chemical Composition, Toxicity, Optical Properties, and Climate Effect. Environ. Int. 2021, 157, 106801. https://doi.org/10.1016/j.envint.2021.106801.

(18) Tuet, W. Y.; Liu, F.; de Oliveira Alves, N.; Fok, S.; Artaxo, P.; Vasconcellos, P.; Champion, J. A.; Ng, N. L. Chemical Oxidative Potential and Cellular Oxidative Stress from Open Biomass Burning Aerosol. Environ. Sci. Technol. Lett. 2019, 6 (3), 126–132. https://doi.org/10.1021/acs.estlett.9b00060.

(19) Wong, J. P. S.; Tsagkaraki, M.; Tsiodra, I.; Mihalopoulos, N.; Violaki, K.; Kanakidou, M.; Sciare, J.; Nenes, A.; Weber, R. J. Effects of Atmospheric Processing on the Oxidative Potential of Biomass Burning Organic Aerosols. Environ. Sci. Technol. 2019, 53 (12), 6747–6756. https://doi.org/10.1021/acs.est.9b01034.
